# Novel polyvinyl alcohol–borax hydrogel with potential to accelerate skin wound healing in aquatic models: zebrafish and tilapia

**DOI:** 10.1098/rsos.252176

**Published:** 2026-09-09

**Authors:** Syuan-Ting Wu, Minh-Quan Tran, Petrus Siregar, Franelyne P. Casuga, Kelvin H.-C. Chen, Szu-Wei Huang, Chih-Hsin Hung, Chung-Der Hsiao

**Affiliations:** ^1^ Institute of Biotechnology and Chemical Engineering, I-Shou University, Kaohsiung, Taiwan; ^2^ Department of Biomedical Engineering, Chung Yuan Christian University, Taoyuan, Taiwan; ^3^ Department of Chemistry, Chung Yuan Christian University, Taoyuan, Taiwan; ^4^ Department of Bioscience Technology, Chung Yuan Christian University, Taoyuan, Taiwan; ^5^ Research Center for Aquatic Toxicology and Pharmacology, Chung Yuan Christian University, Taoyuan, Taiwan; ^6^ Faculty of Pharmacy and The Graduate School, University of Santo Tomas, Manila, Philippines; ^7^ Department of Applied Chemistry, National Pingtung University, Pingtung, Taiwan; ^8^ Department of Veterinary Medicine, National Chiayi University, Chiayi, Taiwan

**Keywords:** zebrafish, tilapia, hydrogel, skin, wound healing

## Abstract

Skin integrity is vital for fish survival, as injuries compromise the protective barrier and increase infection risk. This study introduces a simple and eco-friendly strategy to enhance skin wound healing in aquatic species using a PVA+borax hydrogel. The hydrogel, characterized by tunable cross-linking and biodegradability, was applied to zebrafish and tilapia via water immersion to evaluate its therapeutic efficacy. Full-thickness wounds were created in zebrafish via laser ablation for precise assessment of closure kinetics. Among tested conditions, the PVA+borax formulation significantly accelerated wound closure compared with individual components and untreated controls. Acute toxicity and behavioural analyses confirmed high biocompatibility and no developmental or neurological abnormalities up to higher concentration use (0.01–100 ppm). qRT-PCR analysis showed that treatment reduced injury-induced inflammatory and macrophage markers, suggesting modulation of inflammatory responses during wound healing. Concurrent changes in basement membrane-related genes indicate coordinated extracellular matrix remodelling, consistent with progression towards tissue repair and balanced regeneration. Both zebrafish and tilapia exhibited faster re-epithelialization and collagen deposition under hydrogel treatment. These findings suggest that PVA+borax hydrogel is a safe, cost-effective and promising platform for accelerating wound healing in aquatic models, with translational potential in both biomedical research and aquaculture health management.

## 1. Introduction

Skin wound healing is a tightly regulated, multi-phase process that restores tissue integrity following injury. In vertebrates, this process proceeds through overlapping stages, including inflammation, epithelialization, granulation tissue formation and remodelling [1]. A key regulator is the extracellular matrix (ECM), which provides structural support and governs cellular behaviours such as migration, adhesion and differentiation. Composed of collagen, elastin, laminin and fibronectin, the ECM also acts as a signalling reservoir for cytokines and growth factors that coordinate tissue regeneration [2]. In aquatic animals, wound healing is particularly vulnerable owing to continuous water exposure, which increases infection risk, delays repair and can lead to significant mortality and economic loss in aquaculture. Common injuries, such as scale loss, mucus disruption and abrasions, further enhance susceptibility to microbial infections [3]. In this context, zebrafish are widely used as a preclinical model to investigate fundamental wound-healing mechanisms and evaluate biomaterial safety and efficacy [4], while tilapia serves as a representative farm species for assessing real-world therapeutic potential and scalability in aquaculture applications [5]. Unlike terrestrial systems where treatments, such as debridement, topical agents and hydrogels, are routinely applied [6], aquatic species have limited therapeutic options owing to their water-based environment and fragile skin barrier, highlighting the need for effective biomaterial-based strategies for both preclinical validation and agricultural applications [7]. In this regard, developing biocompatible biomaterials for wound healing has recently attracted more attention.

Hydrogel-based biomaterials have been increasingly explored as a promising platform to overcome these limitations in aquatic wound healing [8], as they are cross-linked polymer networks with high water content that provide a hydrated environment suitable for wound healing [6,7]. Their ability to retain moisture and conform to tissue surfaces makes them effective as wound dressings [9]. Common materials include polyvinyl alcohol (PVA), polyethylene glycol and related synthetic polymers [10,11], among which PVA is widely used owing to its biocompatibility, water solubility and favourable mechanical properties [11]. Compared with natural hydrogels, synthetic hydrogels, such as PVA, offer improved stability and water retention, supporting tissue repair [12]. Accordingly, hydrogels have been widely applied in biomedical fields and shown effectiveness in promoting wound healing in *in vivo* and *in vitro* models (supplementary material, tables S1 and S2) [13]. Despite these advantages, their application in aquatic organisms remains limited. Based on this, we developed a cost-effective PVA+borax hydrogel and evaluated its wound-healing efficacy in aquatic animals. In this system, PVA serves as the structural matrix, while borax acts as a cross-linking agent to enhance hydrogel stability. This study focuses on wound closure and biocompatibility to assess the ability of PVA+borax hydrogel as a practical treatment for skin injuries in aquatic environments.

PVA is a water soluble and biocompatible polyhydroxy polymer known for its non-toxicity, strong hydrophilicity and balanced chemical and mechanical properties [14]. Its moisture absorption and swelling properties make it well-suited for wound-healing applications [15,16]. Owing to these ideal characteristics, PVA is widely used as a synthetic polymer for wound dressings, wound management and drug delivery systems [17,18]. However, PVA has limitations, including insufficient elasticity and a stiff membrane, which can restrict its effectiveness as a wound-healing material [18]. To overcome these drawbacks, PVA is often combined with other compounds such as borax. Borax (Na₂B₄O₇) is a water soluble boron-containing salt [19]. In water, borax anions form boric acid and hydroxide ions, while boronic acid molecules react with hydroxide ions to generate borate ions (BOH₄⁻) [20]. Importantly, boron-based compounds, including borax, have been reported to exhibit antimicrobial activity in experimental studies [21,22], yet their direct use in clinical wound care remains limited. PVA+borax hydrogel is formed by cross-linking PVA and borax in water [23]. PVA enhances water retention, while borax serves as the cross-linking agent. Hydrogen bonds between PVA and borax molecules stabilize the network [24,25]. This combination may contribute to the wound-healing process by providing a favourable environment while preserving antimicrobial and antiseptic activity, indicating PVA+borax hydrogel could be a potential biocompatible and efficient biomaterial for wound-healing applications.

 This study aims to evaluate the wound-healing efficacy and biocompatibility of a PVA+borax hydrogel in aquatic organisms. Mammalian models are traditionally used in wound-healing research owing to their close genetic homology with humans and the availability of established genetic tools [26]; however, their high cost and maintenance demands limit large-scale experimental use. By contrast, aquatic models have emerged as effective alternatives [1] offering advantages, including low cost, ease of maintenance and propagation, optical transparency and strong genetic manipulability [27–30], making them suitable for biomaterial testing and wound-healing studies [1,30]. For preclinical study, zebrafish can potentially serve as a bridge between *in vitro* assays and mammalian *in vivo* systems [31]; however, their use is limited by challenges in drug delivery, as aquatic conditions and small body size make conventional localized treatment approaches difficult. In addition, skin injuries in aquaculture species are a significant concern, often leading to infection, impaired healing and mortality, thereby affecting production sustainability [32–34]. Therefore, this study employs zebrafish as a primary model and tilapia as a representative farmed species to assess the therapeutic potential of the PVA+borax hydrogel, focusing on wound closure healing progression and biocompatibility to determine its applicability in aquatic wound management.

## 2. Material and Methods

### 2.1. Preparation of PVA+borax hydrogel

**Figure 1.**
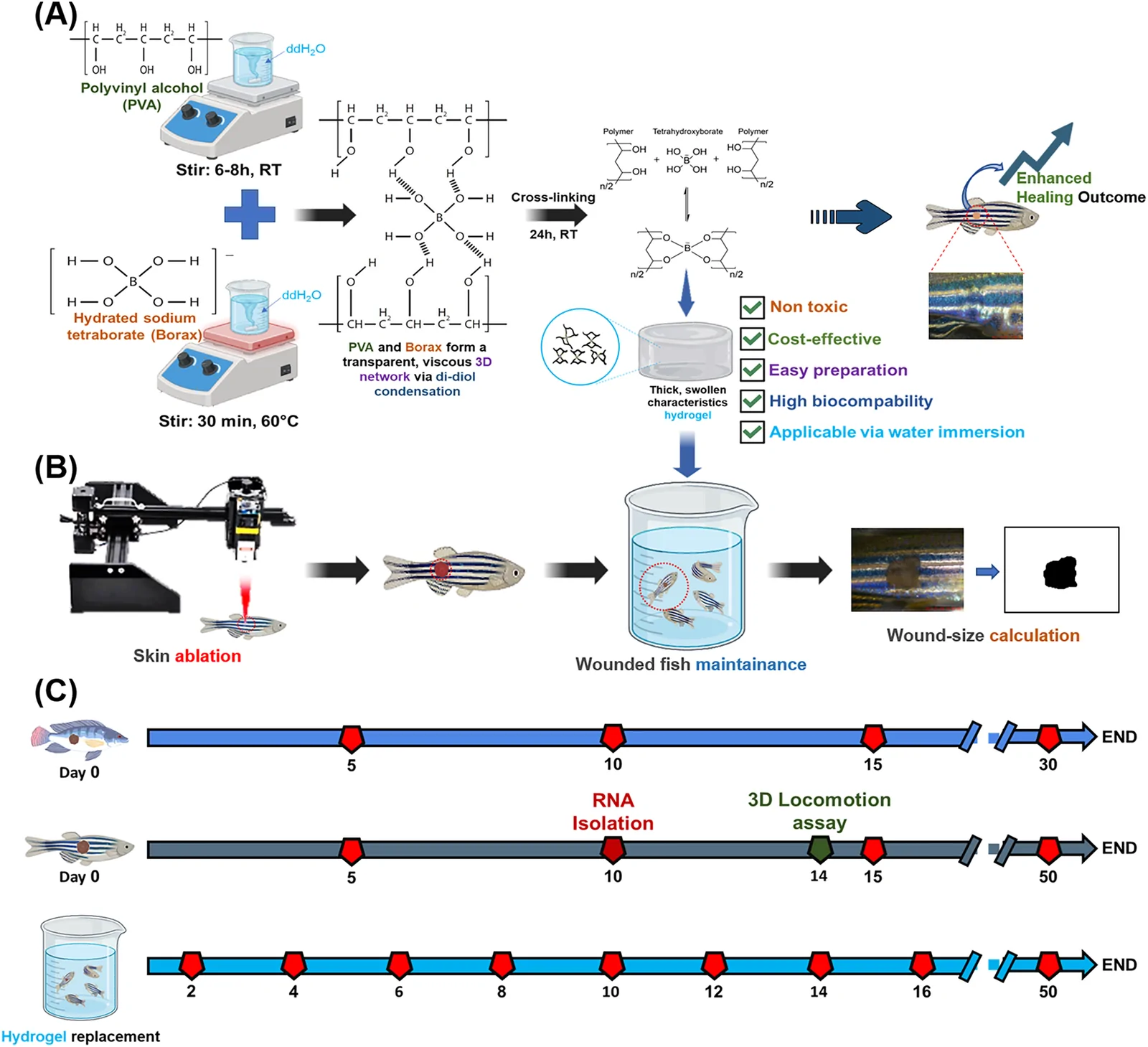
Overview of the experimental design and workflow used to assess the wound-healing effects of hydrogel in fish. (A) Preparation of PVA+borax hydrogel as skin wound rescue material. (B) Laser-based skin ablation assay to evaluate the wound-healing ability of the PVA+borax hydrogel formulation. (C) Experimental timeline for zebrafish and tilapia bioassay of skin wound healing.

The PVA and borax, sourced from Sigma-Aldrich, were used to prepare the PVA+borax hydrogel by dissolving each component separately in precise ratios tailored for various treatments. To ensure the integrity and consistency of the hydrogel, meticulous care was taken during preparation to prevent the formation of air bubbles. Initially, 1.6 g of PVA powder was dissolved in 20 ml of distilled water. The mixture was gently stirred using a magnetic stirrer for 6–8 h at room temperature to achieve a homogenous solution. Meanwhile, 0.8 g of borax powder was dissolved in 20 ml of distilled water at 60°C and stirred for 30 min with a magnetic stirrer to create the borax solution. To prepare the hydrogel, the borax solution was gradually added to the PVA solution. The mixture was gently stirred and then left undisturbed for 24 h to allow the cross-linking reaction between PVA and borax. This reaction forms a hydrogel, as the interaction between PVA and borax molecules creates a network structure (figure 1A). After 24 h, the mixed solution transformed into a hydrogel with thick, swollen characteristics, ready for treatment.

### 2.2. Zebrafish and tilapia maintenance

Zebrafish (*Danio rerio*, PET strain, four to six months old) were obtained from a local pet store and maintained in 10 l plastic tanks under controlled conditions to ensure health and experimental consistency. Water temperature was maintained at 28°C with a 14 h light/10 h dark cycle to mimic the natural circadian rhythm. The system used reverse osmosis-filtered water, with pH maintained between 7.0 and 7.5. Fish were fed twice daily with freshly cultured brine shrimp and premium dry pellets to ensure balanced nutrition.

Tilapia (*Oreochromis aureus*, three to five months old) were also sourced locally and housed in 20 l glass tanks under similar conditions, with water temperature maintained at 28°C and feeding conducted twice daily using commercial dry feed. All experimental procedures were conducted in accordance with the guidelines approved by the Institutional Animal Care and Use Committees (IACUC) of Chung Yuan Christian University (Approval No. 110 016, issued 29 December 2021) [1].

### 2.3. Skin wound-healing assay

This study employed a custom laser-based skin ablation system in zebrafish, adapted from established laboratory protocols [35]. The system integrates a high-power CO₂ laser with an XY motorized stage, enabling precise and reproducible control of wound size and geometry, with reduced invasiveness compared with conventional methods [4,36,37].

Under optimized conditions (3 W; 1 mm s^−1^; 37 s), consistent full-thickness wounds (approx. 2 mm diameter, approx. 400 µm depth) were created on the left flank of four- to six-month-old zebrafish under 0.1% MS-222 anaesthesia, with immobilization in agarose moulds [1]. Fish showed no obvious behavioural changes post-injury. Following the procedure, animals received 5 ppm lidocaine [38] for analgesia and were allowed to recover before being returned to their tanks.

Each experimental group in the wound-healing assay included 10–12 fish, based on prior studies [1,30,35]. This sample size provides sufficient statistical power while aligning with the 3Rs principles, particularly reducing animal use, and is supported by the consistent detection of significant differences across time points. The laser-based method ensures uniform, minimally invasive wounds with precise control, enabling reliable comparisons and evaluation of hydrogel treatment efficacy (figure 1B). In this study, this method was applied to evaluate hydrogel-based treatments in zebrafish models.

### 2.4. Wound-healing visual observation and measurement

Wound healing was systematically monitored from 5 to 50 dpw for zebrafish and from 5 to 30 dpw for tilapia [1]. At each time point, fish were anaesthetized to minimize stress and ensure immobility during imaging. High-resolution images were captured using a digital microscope (Andonstar, Shenzhen, China), providing clear visualization for precise analysis [35]. Individual zebrafish were tracked using unique scale patterns for consistent monitoring. Wound images (.JPG) were analysed with ImageJ (v.1.53k), outlining areas with the free-hand selection tool and measuring via the ROI manager using a calibrated scale. Wound closure percentage (WC%) was calculated from area reduction, with wounds considered fully healed when indistinguishable from surrounding skin in structure and pigmentation (figure 1B). This standardized method provided reliable assessment of healing progression and therapeutic effects, in line with established zebrafish wound-healing protocols [1,35]. All of the images presenting wound-healing progression in zebrafish and tilapia can be found in https://zenodo.org/records/17327603.

### 2.5. Administration of hydrogel as a treatment for injured fish

The prepared hydrogel was subsequently dispersed into system water at defined concentrations to generate the treatment solution and was administered by direct addition to the water tanks containing the experimental fish, following our previously established method [8]. To maintain optimal water quality and prevent contaminant accumulation, the treatment water was refreshed every two days (figure 1C). This approach maintained continuous exposure of the wound to the hydrogel. Under immersion, the PVA+borax hydrogel progressively dissolved (approx. 12–13 h for 20 g hydrogel), enabling sustained release of polymer–borate species (supplementary material, table S4). In FeCl₂ systems, a gradual colour change without precipitation indicated ongoing dispersion and ion interaction (supplementary material, note S1), consistent with reversible borate–diol complexation and Fe²⁺-mediated network dissociation.

Following laser-induced skin ablation, zebrafish were transferred to 2 l tanks containing one of three treatment solutions: PVA, borax or PVA+borax hydrogel. The selection of hydrogel was based on a combination of toxicity screening and functional relevance. Acute embryo toxicity assays showed no mortality at concentrations up to 100 ppm through 120 hpf, indicating a broad safety margin (supplementary material, table S6). Therefore, 2 ppm was selected as a low, sub-effective dose to assess minimal biological response, while 20 ppm represented a higher yet non-toxic concentration expected to elicit measurable therapeutic effects [35]. Similarly, tilapia were maintained in 6 l tanks under the same treatment conditions and concentrations. This standardized design ensured consistent exposure across species, enabling reliable comparative analysis of hydrogel efficacy.

### 2.6. Evaluating the biocompatibility of hydrogel using the morphology assay

Following the identification of representative concentrations, a detailed biosafety assessment was conducted at the highest concentration used in the wound-healing assay (20 ppm), including comprehensive morphological evaluation. After feeding, adult male and female zebrafish were transferred to breeding chambers at a 2:1 ratio. The separator was removed the following morning to initiate spawning, and embryos were collected within 2 hpf. The embryos were gently rinsed with double-distilled water and maintained at 28°C. All samples were standardized to 0 hpf prior to treatment.

Embryos were then allocated into four groups: (i) control, (ii) PVA, (iii) borax, and (iv) PVA+borax hydrogel (supplementary material, figure S1). Morphological development was monitored at 24-h intervals up to 120 hpf. Acute toxicity assessment was conducted in accordance with OECD Test Guideline 236 [39].

### 2.7. Behavioural evaluation of PVA+borax hydrogel in adult zebrafish model

To assess the biocompatibility of the wound-healing agents, adult zebrafish (four to six months old, mixed sex) were exposed to the hydrogel via water immersion at 20 ppm (the highest concentration used for wound-healing applications) and allocated into four experimental groups: (i) control, (ii) PVA-treated, (iii) borax-treated, and (iv) PVA+borax hydrogel–treated. Behavioural assessments were conducted on day 14, following our previous protocol for evaluating biomaterial effects in adult zebrafish [1].

Behavioural analyses were conducted using a novel tank assay with a simplified single-camera three-dimensional tracking system, as described by Audira *et al.* [40]. Six fish were placed in a custom acrylic tank (20 × 20 × 20 cm^3^; water depth approx. 14.5 cm) equipped with a 45° angled mirror to capture multi-angle movement. Video recordings were acquired using a Canon EOS 600D camera (1280 × 720 resolution, 50 fps) fitted with a Canon EF-S 55–250 mm lens, positioned approximately 5–6 m away to minimize distortion, under uniform LED illumination (5500 K) to ensure optimal contrast. Prior to recording, fish were acclimated for 15 min. Locomotor behaviour was tracked using UMATracker, calibrated in ImageJ and further analysed with the Fish 3D Locomotion app (F3LA), a custom software developed for extracting behavioural endpoints (supplementary material, table S3) [41]. This integrated approach enables precise three-dimensional analysis of zebrafish movement, allowing robust evaluation of biomaterial biocompatibility through behavioural performance in a cost-effective and less complex set-up compared with conventional multi-camera systems (supplementary material, figure S1). All of the recorded fish behaviour videos can be found in https://zenodo.org/records/17333407.

### 2.8. Statistical analysis

Statistical analyses were performed using GraphPad Prism (v.8.0.2; GraphPad Inc., La Jolla, CA, USA). Longitudinal wound closure data were analysed using two-way ANOVA with Geisser–Greenhouse correction, followed by Dunnett's post hoc test, which was performed to compare treatment groups with the control. Predicted times to reach 25%, 50% and 75% wound closure were estimated by nonlinear regression, and each treatment group was compared with the control group using unpaired *t*-tests. Gene expression data were analysed using one-way ANOVA followed by Tukey's multiple comparisons test. Behavioural endpoints obtained from the three-dimensional locomotion assay were analysed using the Kruskal–Wallis test with Dunn's multiple comparisons test [1]. All statistical significance was defined as ns: not significant, *p < 0.05, **p < 0.01, ***p < 0.001 and ****p < 0.0001.

### 2.9. RNA isolation

Adult zebrafish were assigned to four experimental groups: (i) healthy control, (ii) injured, (iii) injured with PVA treatment, and **(iv)** injured with borax treatment. All treated groups received the most effective treatment concentration identified from prior optimization experiments. Treatments were administered via immersion in treated water, as outlined in the experimental design (figure 1).

At 10 days post-laser ablation, fish were euthanized by cold-water immersion (2–4°C), and wounded skin tissues were collected for RNA sequencing to investigate global gene expression patterns and associated pathway enrichment. This time point was selected as it corresponds to an active phase of tissue repair, during which differences in healing responses between groups are more evident [1,36]. For each group, three pooled RNA samples were generated, with each sample comprising four tissue specimens. Collected tissues were homogenized in RNAzol® RT (100 mg ml^−1^) using a Bullet Blender. Following homogenization, ddH₂O (0.4 ml per 1 ml RNAzol®) was added, and samples were incubated for 15 min before centrifugation (12 000 rpm, 15 min) to obtain the supernatant.

RNA was subsequently precipitated with isopropanol, pelleted by centrifugation and washed three times with 75% ethanol. The resulting RNA pellet was air-dried and reconstituted in ddH₂O. RNA purity and concentration were evaluated using OD₂₆₀/₂₈₀ ratios (≥2.0), and samples were stored at −20°C until further analysis.

### 2.10. qRT-PCR for gene expression

Quantitative real-time PCR (qRT-PCR) was performed using SYBR Green Master Mix (Protech, Taiwan) on a Bio-Rad Opticon thermal cycler. The amplification programme consisted of an initial denaturation step at 95°C for 2 min, followed by 40 cycles of 95°C for 10 s and 60°C for 30 s, following our previous study protocol [1]. Relative gene expression levels were calculated using the 2^−ΔΔCt^ method and normalized against β-actin as the internal reference gene [1].

A selected panel of zebrafish genes was analysed to evaluate key processes involved in wound healing, including ECM remodelling (*col4a1*, *col4a2*, *mmp9* and *timp2b*), angiogenesis (*vegf*), inflammatory response (*tnfα*, *il1β* and *il10*) and immune regulation (*cd3e*, *cd79a*, *arg2* and *nos2b*). The rationale for gene selection followed a previously established study [1], with details described in table S5 in the supplementary material.

### 2.11. Skin histology

Adult zebrafish receiving hydrogel treatment were fixed in 4% paraformaldehyde/PBS for 1 day and transferred to Davidson's solution (30% ethyl alcohol (95%), 10% acetic acid, 20% formalin and 30% double-distilled water) for 3 days at room temperature. This procedure decalcifies the hard tissues and keeps the skin in good morphology. The decalcified samples were then dehydrated in ascending ethanol, cleared with Neo-Clear (Merck) and embedded in Paraplast Plus/Paraplast HM (Leica) in a 7:3 ratio (vol/vol). The paraffin-embedded tissues were sectioned at 5 µm intervals with a rotational microtome (HM360, Microm) and then stained with an either H&E (TA01HE, BioTnA), Masson's Trichrome (TASS01, BioTnA) or Picrosirius Red (TASS08, BioTnA) staining kits. For immunohistochemistry, CK18 (Peviva, 10650) and PCNA (Bioworld, BS6438) antibodies were used at dilution factor of 1:200 and 1:1000 as first antibody and colour developed with the TnAlink DAB polymer detection system (BioTnA, TAHC04D).

## 3. Results

### 3.1. Skin wound-healing test in zebrafish

The skin wound healing outcomes in zebrafish followed water immersion in PVA, borax and their combined hydrogel formulation at two concentrations (2 and 20 ppm) over 50 dpw. At days 5, 15, 30 and 50, where all groups displayed comparable wound sizes at day 5, the treated groups, particularly at 20 ppm, demonstrated progressively faster wound contraction and scale pattern restoration compared with the untreated control.

**Figure 2.**
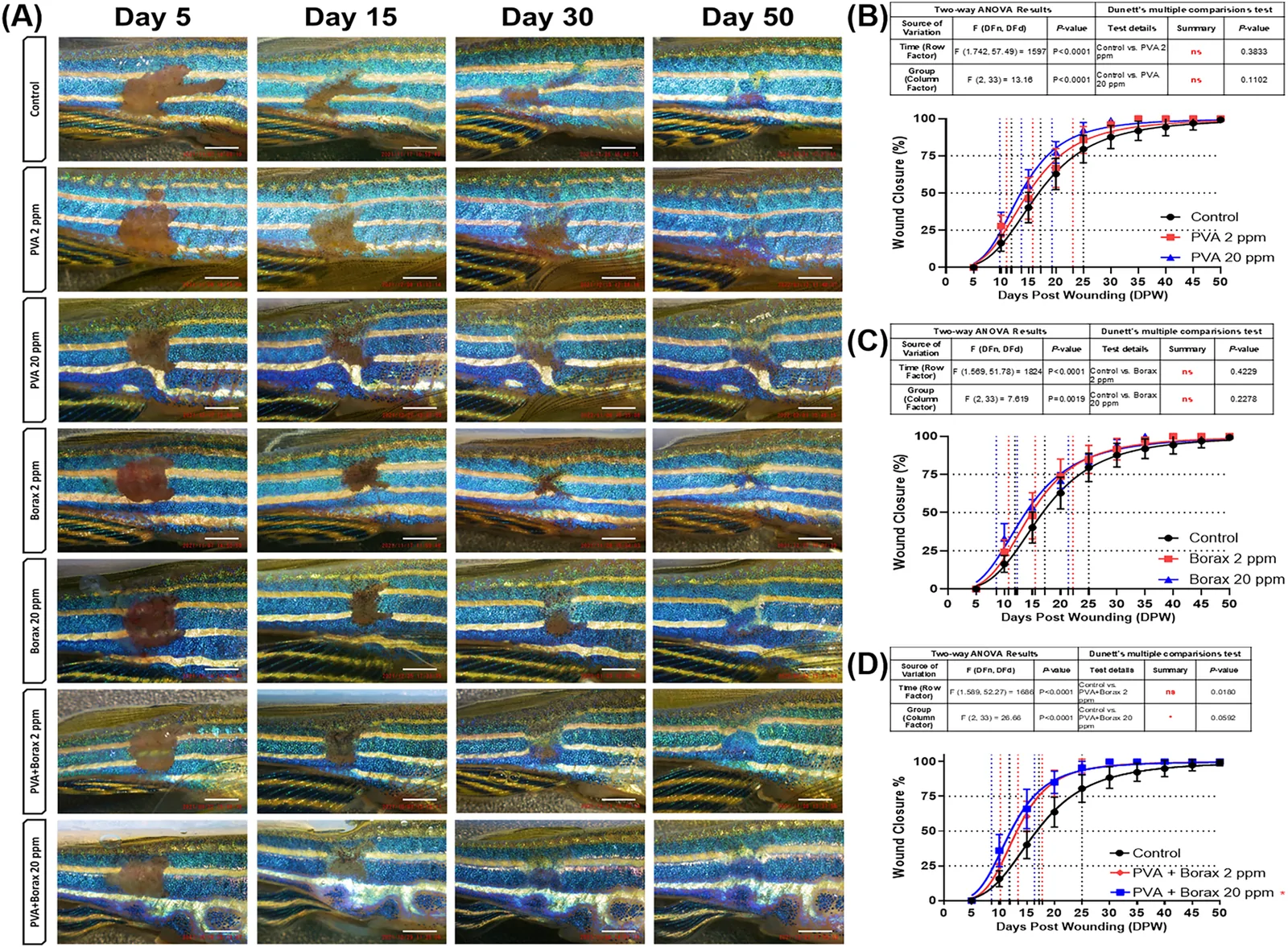
Testing skin wound-healing effect in zebrafish by using hydrogel materials of PVA and borax, single or co-exposure. (A) Representative images presenting wound-healing progression in zebrafish treated with PVA, borax and their hydrogel at either 2 or 20 ppm concentration (scale bar = 1.5 mm). (B) Wound closure percentage over time in zebrafish treated with low (2 ppm) or high (20 ppm) doses of PVA only. (C) Wound closure percentage over time in zebrafish treated with low (2 ppm) or high (20 ppm) doses of borax only. (D) Wound closure percentage over time in zebrafish treated with low (2 ppm) or high (20 ppm) doses of PVA+borax hydrogel. Wound closure data were analysed using two-way ANOVA with Geisser–Greenhouse correction. Dunnett's post hoc test was performed to compare each treatment group with the control. Data presented as mean ± s.d., with sample sizes of *n* = 10–12. Statistical significance is defined as ns: not significant and *p < 0.05.

Quantitative analysis of wound closure (figure 2; table 1) demonstrated a clear dose-dependent improvement across treatments. At 20 ppm, PVA significantly reduced the time to reach 25%, 50% and 75% closure (9.8 ± 1.9, 13.7 ± 1.9 and 19.3 ± 2.4 days, respectively; table 1) compared with the control (11.9 ± 1.8, 17.2 ± 2.4 and 25.1 ± 4.8 days). Similarly, borax at 20 ppm further accelerated early healing, reaching 25% and 50% closure at 8.6 ± 1.7 and 12.3 ± 3.9 days. Notably, the PVA+borax hydrogel produced the strongest improvement among the tested treatments, significantly shortening closure times at both 2 and 20 ppm. At 20 ppm, wounds reached 25%, 50% and 75% closure at 8.6 ± 1.4, 11.8 ± 1.8 and 16.4 ± 2.8 days, respectively, indicating a pronounced complementary effect compared with individual treatments and control. The combined comparative wound closure kinetics across different treatments are presented in figure S2 in the supplementary material.

**Table 1.** Comparison of skin wound closure between control and PVA, borax and PVA+borax hydrogel-treated groups for zebrafish.

groups	25% wound closure prediction (days)			50% wound closure prediction (days)			75% wound closure prediction (days)		
control	11.9 ± 1.8	F, DFn, Dfd	*p*-value	17.2 ± 2.4	*F*, DFn, Dfd	*p*-value	25.1 ± 4.8	F, DFn, Dfd	*p*-value
**PVA 2 ppm**	11 ± 3.2	3.130, 11, 11	0.4009	15.8 ± 3.1	1.745, 11, 11	0.2349	23.1 ± 4.3	1.217, 11, 11	0.2971
**PVA 20 ppm**	9.8 ± 1.9**	1.104, 11, 11	0.0096	13.7 ± 1.9***	1.408, 11, 11	0.0007	19.3 ± 2.4**	4.036, 11, 11	0.0011
**borax 2 ppm**	10.8 ± 2.1	1.371, 11, 11	0.1863	15.5 ± 3.2	1.784, 11, 11	0.1426	22.2 ± 4.8	1.241, 11, 11	0.1785
**borax 20 ppm**	8.6 ± 1.7***	1.189, 11, 9	0.0002	12.3 ± 3.9**	2.868, 11, 11	0.0013	21.4 ± 4.1	1.355, 11, 11	0.0571
**PVA+borax 2 ppm**	10.2 ± 1.5*	1.531, 11, 11	0.0158	13.4 ± 1.8***	1.786, 11, 11	0.0002	17.8 ± 2.3****	4.403, 11, 11	<0.0001
**PVA+borax 20 ppm**	8.6 ± 1.4****	1.811, 11, 11	<0.0001	11.8 ± 1.8****	1.785, 11, 11	<0.0001	16.4 ± 2.8****	2.952, 11, 11	<0.0001

*Note:* Predicted wound closure times at 25%, 50% and 75% were estimated by nonlinear regression and compared using unpaired *t*-tests. Statistical significance is defined as *p < 0.05, **p < 0.01, ***p < 0.001 and ****p < 0.0001 versus the control group.

These findings suggest that combining PVA and borax as a hydrogel system resulted in greater wound-healing efficacy than either component alone under the tested conditions. The improved performance of the cross-linked hydrogel formulation may reflect the complementary properties of its individual components, with a dose-dependent advantage observed at the higher concentration.

### 3.2. Skin wound-healing test in tilapia

The wound-healing evaluation in tilapia was conducted using the same treatment groups, PVA, borax and PVA+borax hydrogel at 2 and 20 ppm. In tilapia, only a 30-day observation period was required under the optimized laser set-up (3W). At days 5, 15, 30 and 50, when tilapia wounds appeared more irregular than those in zebrafish, reflecting the greater body mass of this species, yet a visible trend towards faster tissue re-epithelialization and scale regeneration was observed in hydrogel-treated fish, particularly at 20 ppm, by day 30.

**Figure 3.**
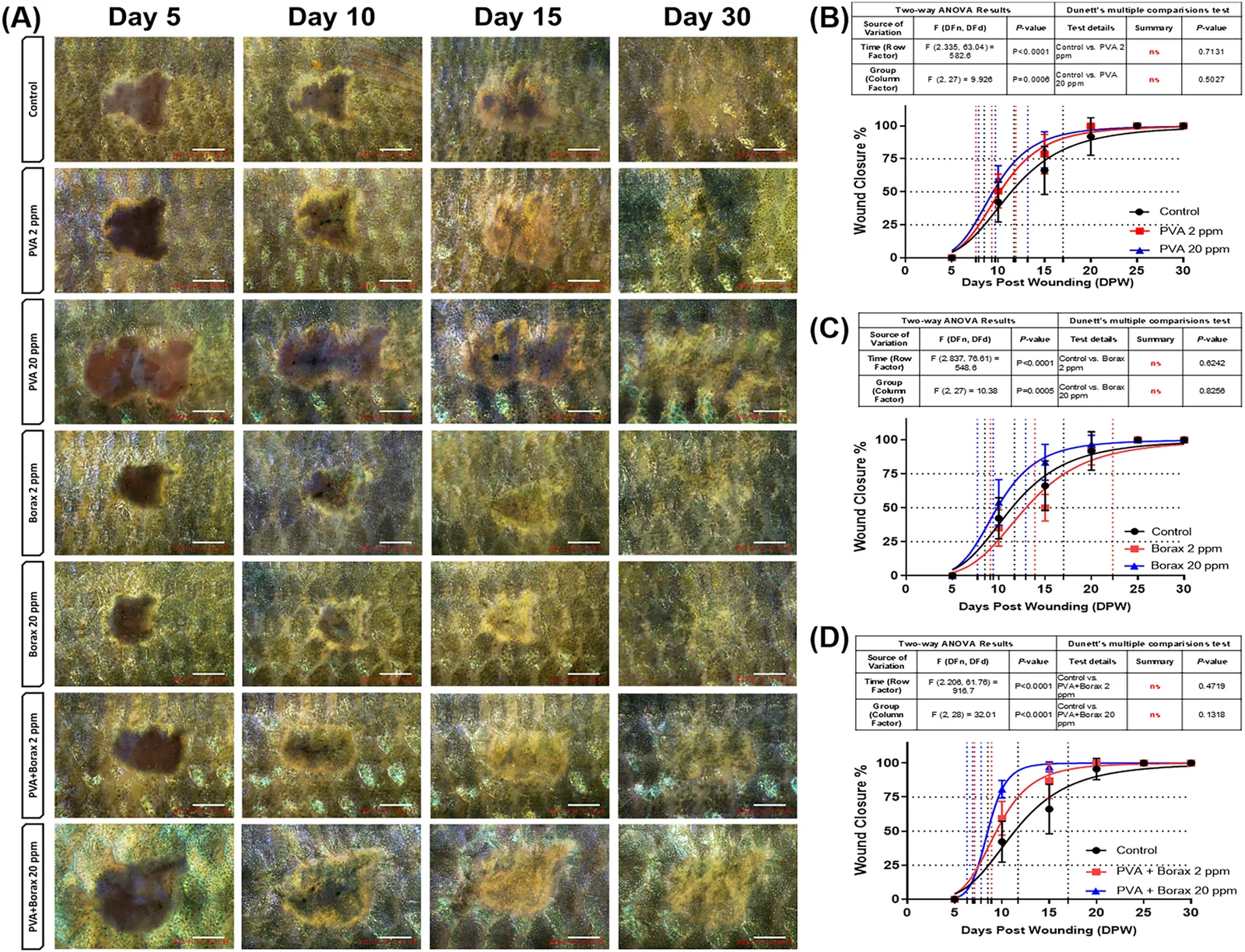
Testing skin wound-healing effect in tilapia by using hydrogel materials of PVA and borax, single or co-exposure. (A) Representative images presenting wound-healing progression in tilapia treated with PVA, borax and their hydrogel at either 2 or 20 ppm concentration (scale bar = 1.5 mm). (B) Wound closure percentage over time in tilapia treated with either low (2 ppm) or high (20 ppm) doses of PVA. (C) Wound closure percentage over time in tilapia treated with either low (2 ppm) or high (20 ppm) doses of borax. (D) Wound closure percentage over time in tilapia treated with either low (2 ppm) or high (20 ppm) doses of PVA+borax hydrogel. Wound closure data were analysed using two-way ANOVA with Geisser–Greenhouse correction. Dunnett's post hoc test was performed to compare each treatment group with the control. Data presented as mean ± s.d., with sample sizes of *n* = 10–11. Statistical significance is defined as ns: not significant.

Consistent with zebrafish results, wound healing in tilapia (figure 3; table 2) was enhanced by hydrogel treatment, with the most pronounced effects observed at 20 ppm. While PVA alone showed limited improvement, only significantly reducing the 75% closure time (11.9 ± 2.3 days versus control at 17 ± 4.7 days), and borax showed minimal or inconsistent effects, the combined hydrogel treatment markedly accelerated healing. At 20 ppm, the PVA+borax group reached 50% and 75% closure at 6.9 ± 3.5 and 7.8 ± 3.1 days, respectively, compared with 11.7 ± 2.8 and 17 ± 4.7 days in controls (table 2). Even at 2 ppm, the hydrogel significantly improved healing at 50% and 75% closure (8.9 ± 2.0 and 11.7 ± 2.8 days), supporting its superior efficacy and translational relevance in aquaculture models. The combined comparative wound closure kinetics across different treatments are presented in figure S3 in the supplementary material.

**Table 2.** Comparison of skin wound closure between control and PVA, borax and PVA+borax hydrogel-treated groups for tilapia.

**groups**	25% wound closure prediction (days)			50% wound closure prediction (days)			75% wound closure prediction (days)		
control	8.5 ± 3.4	F, DFn, Dfd	*p*-value	11.7 ± 2.8	F, DFn, Dfd	*p*-value	17 ± 4.7	F, DFn, Dfd	*p*-value
**PVA 2 ppm**	7.9 ± 1.4	6.101, 8, 7	0.6822	9.7 ± 2.5	1.204, 8, 8	0.1154	13.2 ± 3.4	1.938, 8, 8	0.0603
**PVA 20 ppm**	7.6 ± 2.4	2.070, 8, 6	0.5474	9.3 ± 1.6	2.855, 8, 6	0.0639	11.9 ± 2.3*	4.239, 8, 6	0.0201
**borax 2 ppm**	9.1 ± 3.5	1.042, 9, 8	0.7048	13.9 ± 2.4	1.407, 8, 9	0.0764	22.3 ± 5.5*	1.387, 8, 8	0.0422
**borax 20 ppm**	7.7 ± 1.9	3.137, 8, 8	0.5279	9.4 ± 2.3	1.303, 8, 9	0.0693	12.9 ± 4.2	1.232, 8, 9	0.0613
**PVA+borax 2 ppm**	7.1 ± 2.5	1.884, 8, 10	0.2984	8.9 ± 2*	1.898, 8, 10	0.0154	11.7 ± 2.8**	2.861, 8, 9	0.0066
**PVA+borax 20 ppm**	6.3 ± 3.7	1.165, 9, 8	0.2051	6.9 ± 3.5**	1.572, 9, 8	0.0046	7.8 ± 3.1****	2.342, 8, 9	<0.0001

*Note:* Predicted wound closure times at 25%, 50% and 75% were estimated by nonlinear regression and compared using unpaired *t*-tests. Statistical significance is defined as *p < 0.05, **p < 0.01 and ****p < 0.0001 versus the control group.

Nevertheless, the directional trend consistently mirrors the zebrafish findings; the hydrogel formulation outperformed both individual components across both species, supporting the broader applicability and translational relevance of the PVA+borax hydrogel as a wound care strategy in aquatic animal models.

### 3.3. Immune and inflammatory responses in zebrafish wound healing under treatment

**Figure 4.**
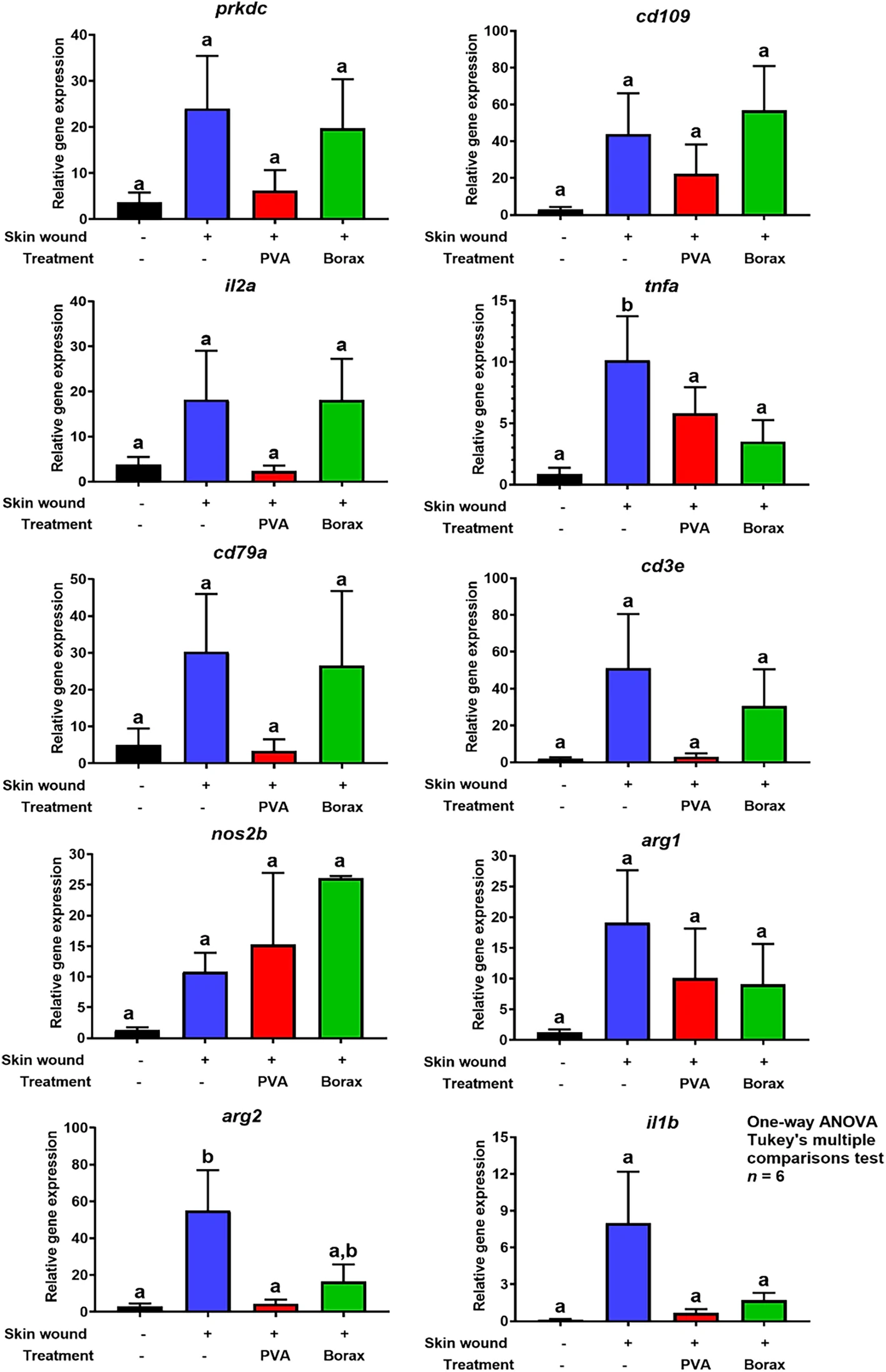
Expression of immune and inflammatory genes in zebrafish skin wounds treated with either PVA or borax. Gene expression was assessed by RT-qPCR and normalized to reference genes. Experimental groups included uninjured controls (black bars), wounded fish without treatment (blue bars), PVA-treated wounds (red bars) and borax-treated wounds (green bars). Data are presented as mean ± SEM (*n* = 6). Statistical significance was determined using one-way ANOVA followed by Tukey's multiple comparisons test. Different letters (a, b) indicate statistically significant differences (*p* < 0.05).

At 10 dpw, the expression of immune- and inflammation-related genes was assessed by qRT-PCR in zebrafish skin (figure 4). Compared with uninjured controls, the wounded group showed increased transcript levels of several genes, including *tnfa*, *il1b*, *cd3e*, *cd79a*, *nos2b* and *arg2*, indicating activation of immune-associated gene expression at the injury site. Among the genes analysed, *tnfa* showed a clear difference between groups; its expression was elevated in the wounded group relative to controls, whereas both PVA- and borax-treated groups exhibited lower expression levels compared with the wounded group; this trend may indicate a reduction in pro-inflammatory gene expression under treatment conditions. A similar trend was observed for *il1b* and *cd3e*, where expression was higher in untreated wounds and lower in the PVA-treated group, with intermediate levels in the borax-treated group. By contrast, *nos2b* expression appeared higher in both treatment groups compared with the wounded group, with the highest mean values observed in the borax-treated fish. This may reflect differences in nitric oxide-related responses. For *arg2*, expression was significantly elevated in the wounded group compared with controls, reduced in the PVA-treated group to levels comparable to the control, while borax-treated fish exhibited intermediate expression, suggesting differential regulation of macrophage-associated responses. Overall, these data indicate that wound induction alters immune-related gene expression and that PVA and borax treatments are associated with distinct transcriptional profiles.

### 3.4. Regenerative potential of hydrogel component treatment

**Figure 5.**
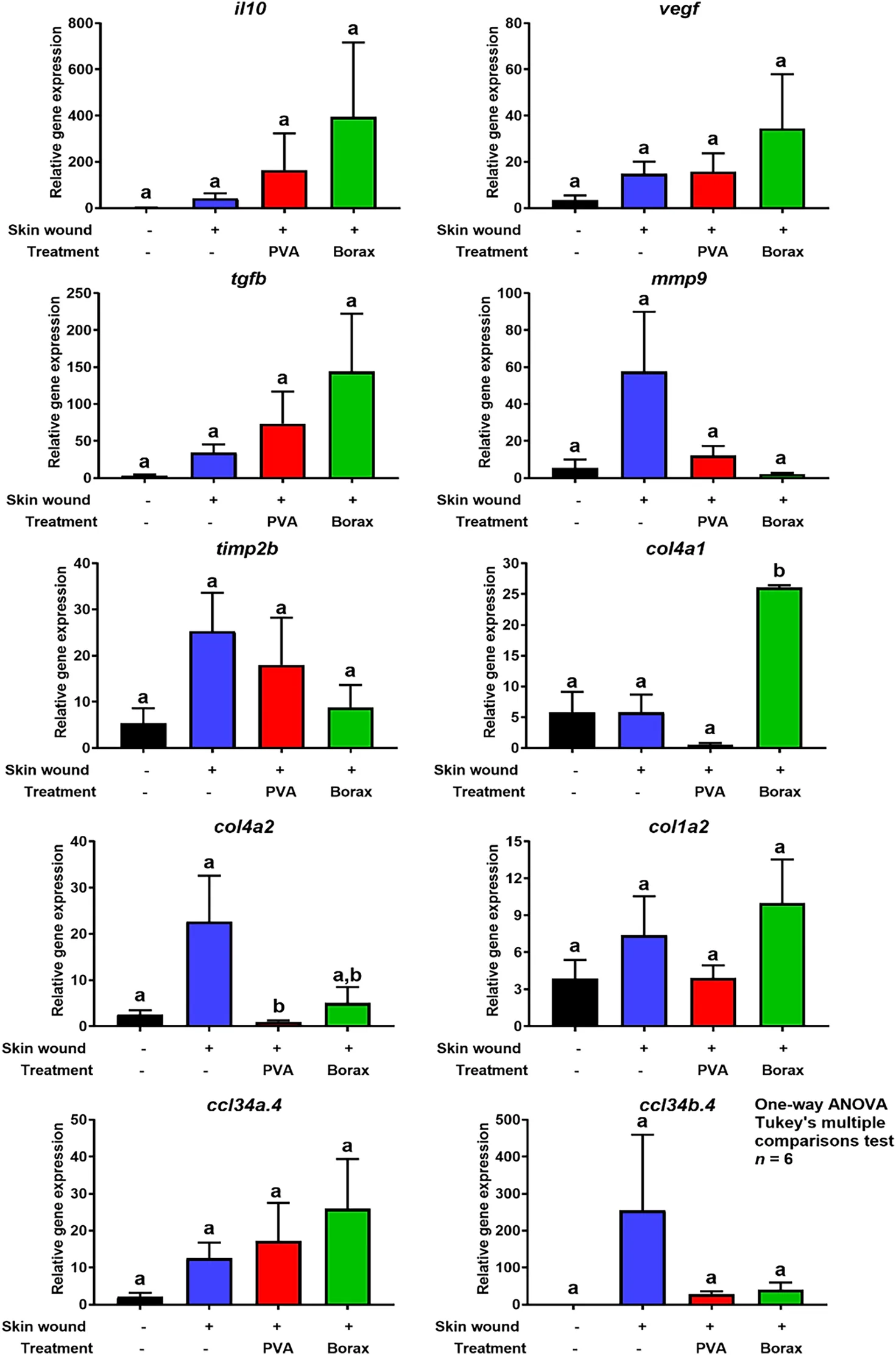
Expression of angiogenesis and ECM-related genes in zebrafish skin wounds treated with either PVA or borax. Experimental groups included uninjured controls (black bars), wounded fish without treatment (blue bars), PVA-treated wounds (red bars) and borax-treated wounds (green bars). Data are presented as mean ± SEM (*n* = 6). Statistical significance was determined using one-way ANOVA followed by Tukey's multiple comparisons test. Different letters (a, b) indicate statistically significant differences (*p* < 0.05).

The expression of genes associated with angiogenesis and ECM remodelling was evaluated by qRT-PCR in zebrafish wound tissue (figure 5). Compared with uninjured controls, the wounded group showed increased expression of several genes, including *vegf*, *tgfb1* and *il10*, indicating activation of repair-associated transcriptional responses following injury. Both PVA- and borax-treated groups exhibited comparable or slightly higher expression levels of *vegf* and *tgfb1* relative to the wounded group, with borax-treated fish generally showing higher mean values; this may reflect modest changes in pro-repair gene expression, potentially in angiogenesis and growth factor-related pathways. For ECM-related genes, *mmp9* expression was elevated in the wounded group and appeared reduced in both treatment groups towards levels closer to controls; this may indicate a shift in matrix remodelling dynamics as healing progresses. A similar trend was observed for *timp2b*. The chemokine *ccl34b.4* was strongly upregulated in the wounded group and showed lower expression in both treatment groups. By contrast, *ccl34a.4* and *col1a2* displayed gradual increases following wounding without clear differences between treatments, which may indicate limited responsiveness under the present conditions. Importantly, both *col4a1* and *col4a2* showed significant differences between groups. *col4a1* expression was elevated in the borax-treated group compared with the wounded group, while *col4a2* expression was reduced in both treatment groups, with a more pronounced decrease in the PVA-treated group. As both genes encode type IV collagen components, these findings indicate that PVA and borax differentially regulate collagen IV-related gene expression, indicating distinct effects on basement membrane remodelling during wound healing. Overall, these results indicate that wound induction and treatment conditions are associated with changes in the expression of genes related to wound-healing progression.

### 3.5. Histological assay of wounded zebrafish under hydrogel treatment

**Figure 6.**
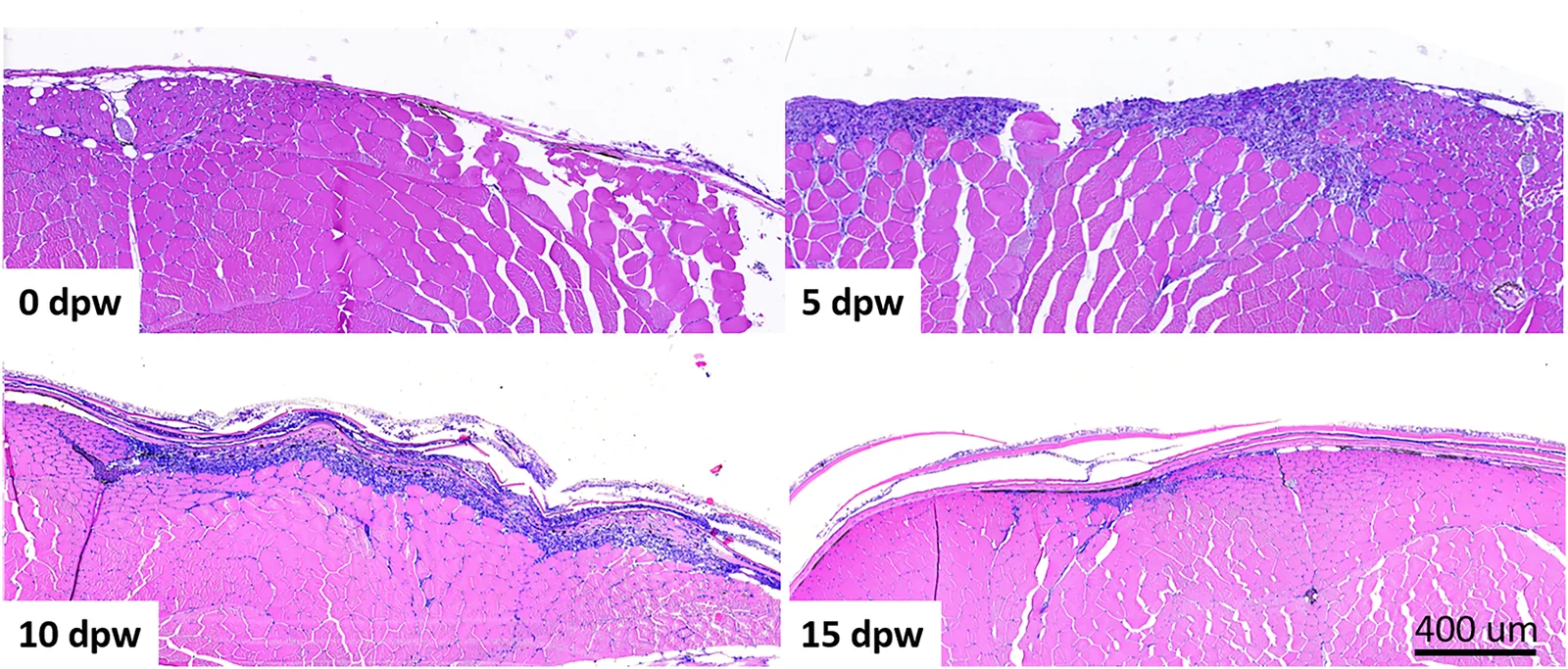
H&E-stained histology image of zebrafish wound ablation until 15 dpw. dpw, day post-wound. Scale bar = 400 µm.

Histological analysis using H&E staining was performed to validate the reproducibility and effectiveness of the laser-based skin ablation system in zebrafish (figure 6). The optimized laser parameters consistently generated a well-defined full-thickness wound of approximately 2 mm width and approximately 0.4 mm depth, confirming, in our previous study, the focus on optimizing the laser set-up, sufficient to trigger a complete wound-healing response [35].

Across the time course (0–15 dpw), histological sections showed a reproducible healing sequence (figure 6). At 5 dpw, the wound area exhibited disrupted epidermis with early re-epithelialization and migration of epidermal cells. By 10 dpw, a continuous but immature neo-epidermis covered the wound bed, indicating ongoing proliferative repair. At 15 dpw, further epidermal consolidation and improved tissue organization were observed, consistent with progression towards the remodelling phase. Overall, H&E staining confirmed that the laser ablation system reliably produces consistent wounds and allows clear visualization of sequential skin-healing stages in zebrafish under hydrogel treatment.

At 5 dpw, disrupted epidermal structure and early keratinocyte migration were observed, consistent with the inflammatory phase and increased expression of *tnfa* and *il1b* (figure 4). By 10 dpw, the formation of a continuous neo-epidermal layer indicated active re-epithelialization, matching the reduced wound area and modulation of inflammatory and ECM-related genes such as *mmp9* and *timp2b* (figure 5). At 15 dpw, improved epidermal continuity and dermal organization were evident, corresponding to near-complete wound closure in treated groups (figures 2 and 3). Overall, the histological findings are consistent with progressive tissue repair that corresponds with the macroscopic and molecular observations.

**Figure 7.**
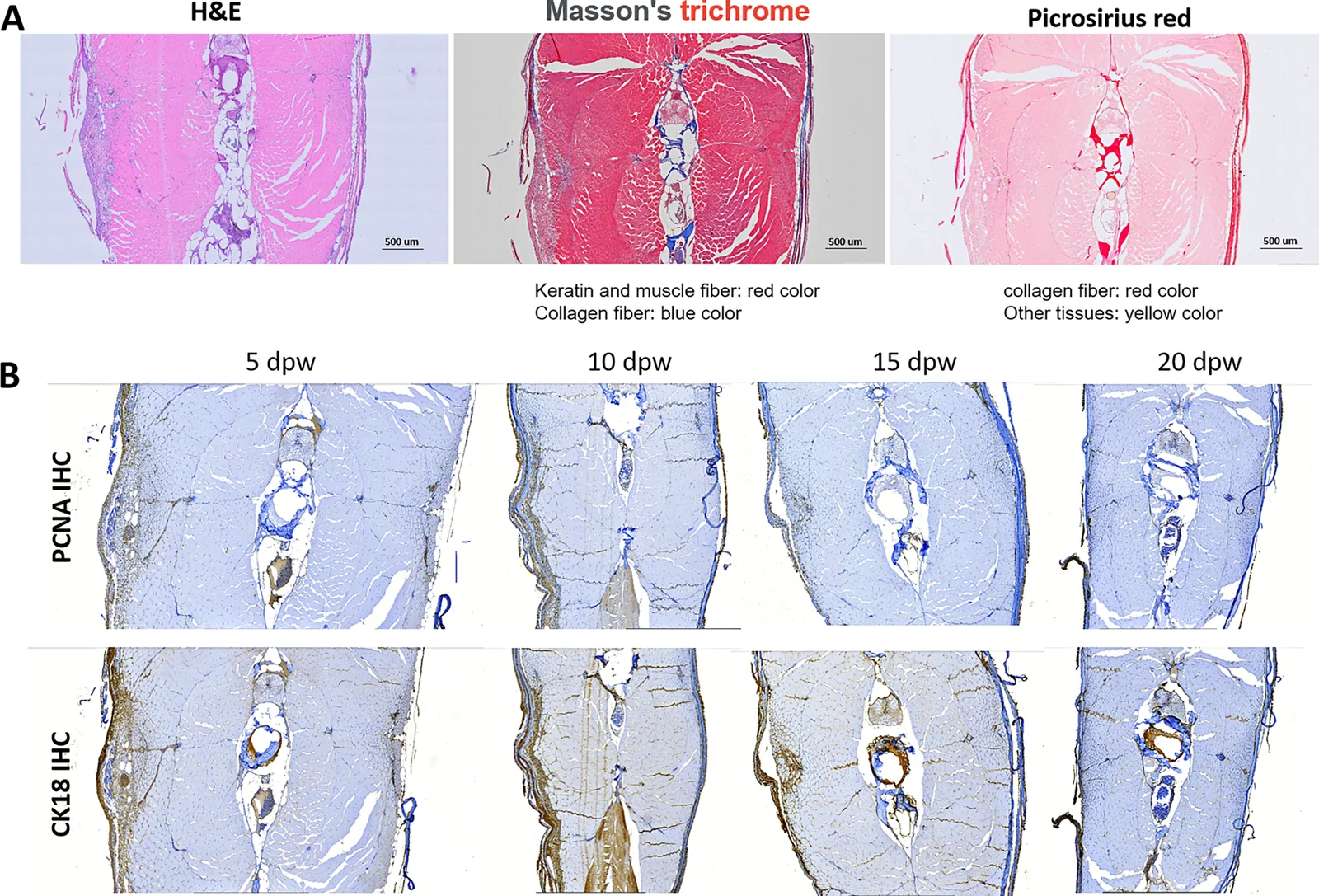
Immunohistochemical results of wounded zebrafish over 20 days. (A) Result of H&E, Masson's trichome and picrosirius red of wound zebrafish on day 5. (B) Result for the PCNA and CK18 immunostaining of wound zebrafish from day 5 through day 20. The left side of fish trunk is the wound area, and the right side is the unwounded control. dpw, day post-wound. Scale bar = 500 µm.

To further elucidate the cellular mechanisms underlying accelerated wound healing, immunohistochemical staining was performed to evaluate proliferation and differentiation markers during tissue regeneration (figure 7). These results complement the gene expression profiles (figures 4 and 5) and are consistent with the histological and macroscopic wound closure patterns shown in wound-healing outcomes. At 5 dpw, histological analysis revealed a disrupted wound surface with an early neo-epidermal layer migrating across the injury site, while the tissue remained loosely organized with incomplete dermal coverage, indicating an early re-epithelialization stage (figure 7A).

Consistently, PCNA showed strong expression at 5 dpw in the wound region (figure 7B), indicating active cell proliferation, which aligns with elevated immune- and repair-related gene expression (figure 4) and the initial reduction in wound area (figures 2 and 3). By 10–15 dpw, PCNA expression progressively decreased towards baseline, reflecting reduced proliferative activity as tissue repair advanced, in agreement with the downregulation of inflammatory genes such as *tnfa* and *il1b* (figure 4) and normalization of ECM remodelling markers, including *mmp9* and *timp2b* (figure 5). By contrast, CK18 exhibited sustained expression throughout 5–15 dpw, indicating continuous epithelial differentiation and maturation, which corresponds with improved tissue organization (figure 7A) and near-complete wound closure in hydrogel-treated groups (figure 2). Overall, the temporal shift from high PCNA to sustained CK18 expression reflects a coordinated transition from proliferation to differentiation, integrating molecular, histological and functional evidence consistent with accelerated wound closure under hydrogel treatment.

### 3.6. Biosafety assay of PVA+borax hydrogel in zebrafish embryos

A toxicity assay was conducted using zebrafish embryos from 0 to 120 hpf to evaluate the biocompatibility of the PVA+borax hydrogel. Embryos were exposed to concentrations ranging from 0.01 to 100 ppm and assessed every 24 h for survival, hatching rate and morphological development, with treatment solutions refreshed at each observation point. No mortality was observed in any group, indicating 100% survival across all tested concentrations (supplementary material, table S6), which supports the biocompatibility of the hydrogel under the tested conditions.

**Figure 8.**
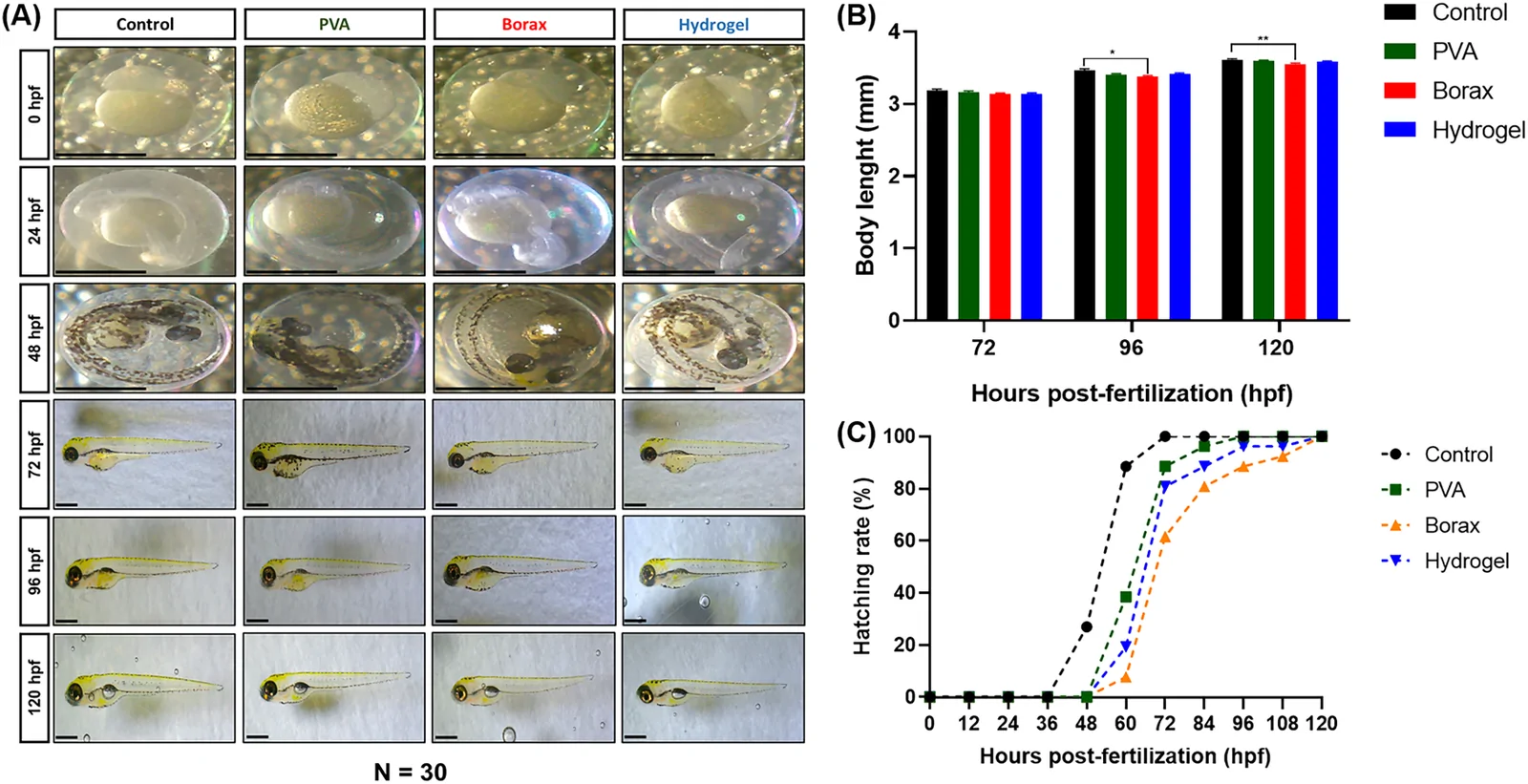
Biocompatibility assessment of PVA, borax and their hydrogel mixture. (A) Representative morphological observations of zebrafish larvae aged from 24 to 120 hpf. (B) Body length analysis of larvae aged at 72, 96 and 120 hpf. Data are presented as mean ± SEM and were analysed using unpaired *t*-tests comparing each treatment group with the control group (*n* = 30; *p < 0.05 and **p < 0.01). (C) Hatching percentages following 0–120 hpf (*n* = 30). Scale bar = 600 µm.

Morphological analysis further showed that embryos treated with PVA, borax or the hydrogel at 20 ppm (the highest concentration used in the wound-healing assay) exhibited normal eye development, body shape and tail formation, comparable to untreated controls (figure 8A). However, hatching was delayed across all treatment groups relative to controls, following the trend: PVA < hydrogel < borax. Notably, borax alone caused the most pronounced developmental delay (figure 8C), with significantly reduced body length from 72 to 120 hpf compared with the control, PVA and hydrogel groups (figure 8B). These findings indicate that while borax alone negatively affects embryonic development, PVA is highly biocompatible, and its incorporation into the hydrogel mitigates borax-associated toxicity. Based on this biosafety profile, 2 and 20 ppm were selected as representative low and high concentrations for subsequent bioassays, including wound-healing studies [35].

### 3.7. Behavioural alterations in zebrafish under the following treatment

Behavioural effects of hydrogel treatment were evaluated using a three-dimensional locomotion assay across four groups: control, PVA-treated, borax-treated and hydrogel-treated zebrafish at 20 ppm. Fourteen behavioural endpoints were assessed and grouped into locomotor activity, movement orientation, movement complexity (fractal dimension and entropy) and exploratory behaviour.

In the locomotor activity domain, hydrogel-treated fish exhibited average speed, rapid movement time and swimming duration (supplementary material, figure S4A–D) comparable to controls, whereas PVA and borax groups showed more pronounced deviations. In the movement orientation domain, meandering and angular velocity (supplementary material, figure S4E–F) were slightly altered in hydrogel-treated fish but remained close to control levels, indicating minimal disorientation. Movement complexity parameters (supplementary material, figure S4G–H) showed no significant differences among groups. Exploratory behaviour (supplementary material, figure S4I–N) revealed mild shifts in zone preference across treated groups, suggesting minor anxiety-like responses.

**Figure 9.**
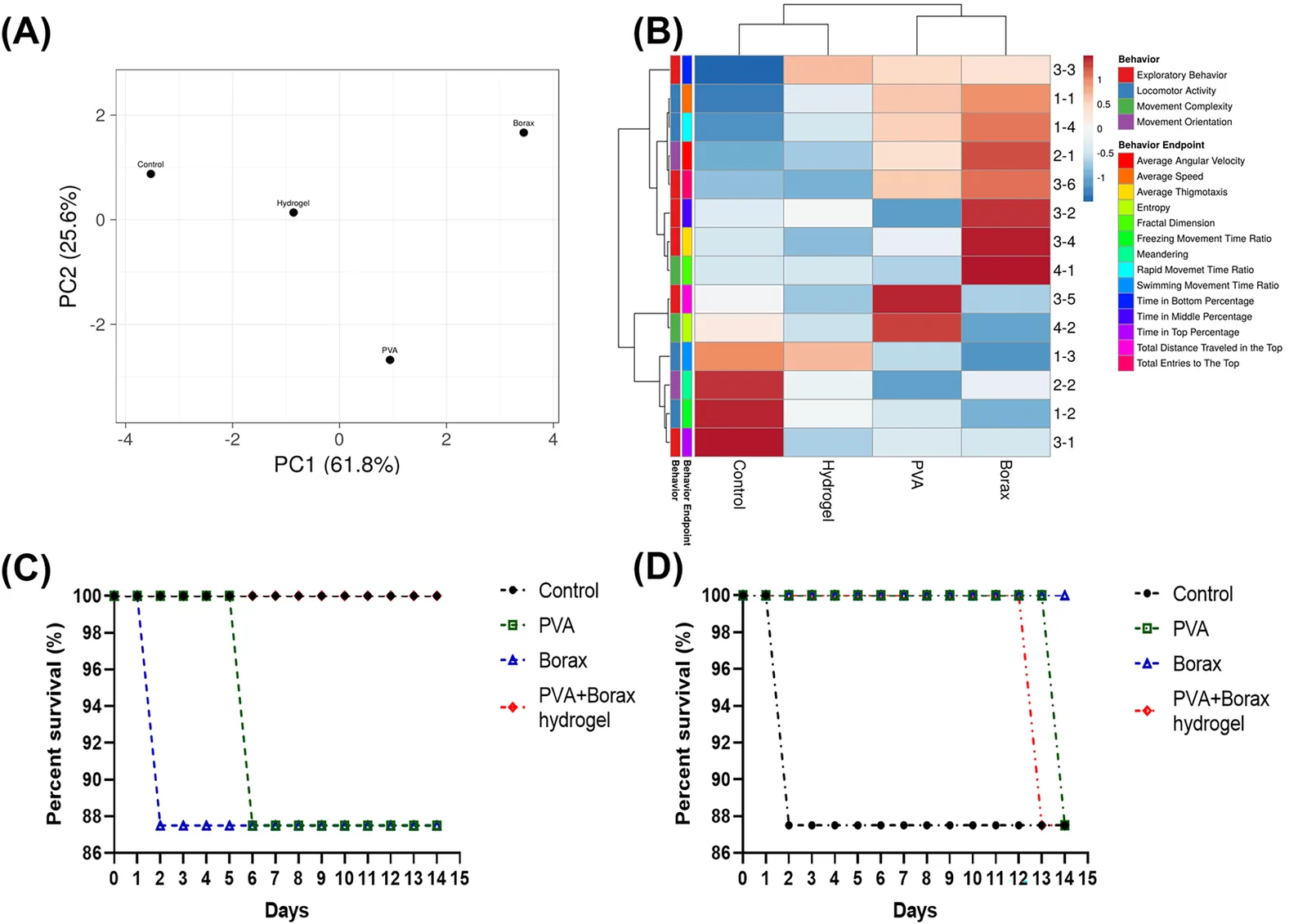
Behavioural analysis of adult zebrafish in a three-dimensional locomotion test after exposure to either PVA, borax or PVA+borax hydrogel. (A) Principal component analysis (PCA) and (B) hierarchical clustering analysis of behavioural activity in different treatment groups and survival rates of adult zebrafish across different treatment groups, shown for (C) the first trial and (D) the second trial (*n* = 14).

Multivariate analyses supported these observations. Principal component analysis (figure 9A) clustered hydrogel-treated fish closely with controls, while PVA and borax groups were clearly separated along PC1, reflecting greater behavioural disruption. Hierarchical clustering (figure 9B) similarly grouped hydrogel-treated fish with controls, distinct from PVA and borax, which exhibited reduced locomotor and complexity-related performance.

**Figure 10.**
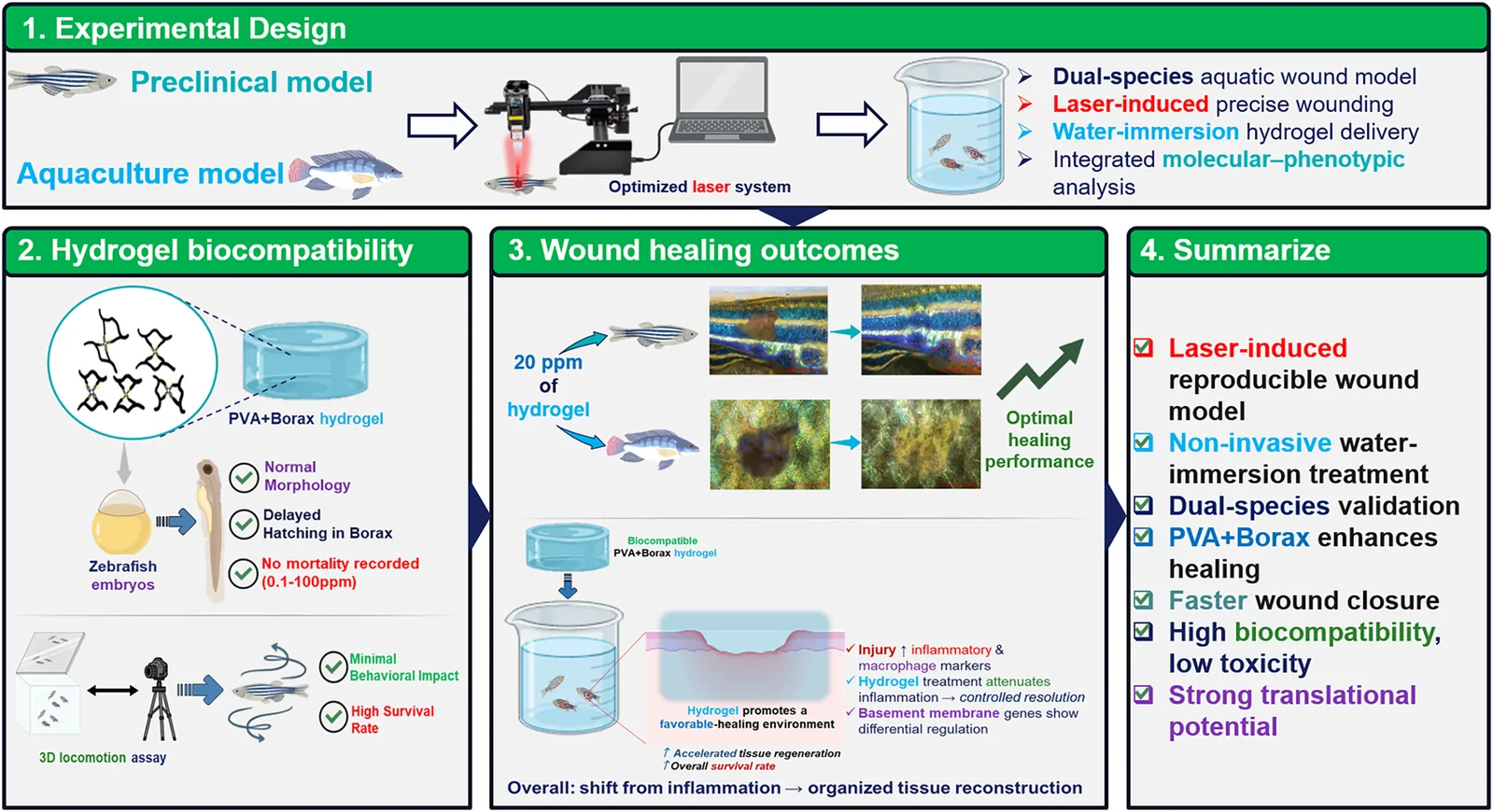
Schematic summary of wound-healing outcomes and therapeutic effects of PVA+borax hydrogel in zebrafish. A standardized dual-species workflow using zebrafish (preclinical) and tilapia (aquaculture) was established, where reproducible wounds were created with an optimized laser system and treated via non-invasive water immersion hydrogel delivery, followed by integrated molecular and phenotypic analyses. Biocompatibility results showed that PVA+borax hydrogel is well tolerated, with normal embryonic morphology, delayed hatching under borax exposure, no mortality at 0.1–100 ppm and minimal behavioural effects with high survival in adults. At 20 ppm, the hydrogel enhanced tissue regeneration and promoted more organized repair in both species, likely by modulating inflammation and supporting extracellular matrix remodelling. Overall, this model demonstrates reproducible injury, effective non-invasive treatment, improved wound closure, high biocompatibility, low toxicity and promising translational potential.

Notably, at this concentration of hydrogel, only minor behavioural alterations were observed in hydrogel-treated zebrafish, primarily manifested as mild anxiety-like responses. As this concentration represents the upper range typically applied in wound-healing applications, these findings suggest that higher doses may induce more pronounced behavioural perturbations. Nevertheless, the hydrogel maintained strong biocompatibility and bioefficacy (figures 2 and 3) while minimizing chemical-induced stress even at this upper therapeutic limit. Overall, the PVA+borax hydrogel-treated fish displayed behavioural profiles that more closely resembled the control group than fish exposed to the individual components, demonstrating a largely neutral behavioural profile, high biocompatibility and low mortality (figure 9C,D), supporting its safety for biomedical wound-healing applications. The summary of the current study can be found in figure 10.

## 4. Discussion

### 4.1. Aquatic animal models for wound-healing study

Aquatic animal models have become valuable alternatives for studying wound healing and testing biomaterial-based therapies. Traditionally, mammalian models, such as rodents, are widely used owing to their similarity to humans, their high cost, ethical concerns and limited scalability restrict broader use [42]. By contrast, zebrafish offer advantages, including low cost, rapid reproduction and optical transparency, supporting their growing role in preclinical research [1,30]. Zebrafish have been widely used to study tissue regeneration owing to their strong capacity for rapid skin repair with minimal scarring [1,36]. In addition, their sensitivity to environmental and chemical perturbations and compatibility with high-throughput analysis make them suitable for evaluating biomaterial-based treatments (figures 2, 8, 9) [8]. Moreover, conserved pathways in cytokine signalling, angiogenesis and collagen deposition highlight their translational relevance to mammalian systems [1].

In this study, zebrafish and tilapia were used as complementary models to evaluate hydrogel-based wound healing in aquatic environments. Zebrafish provided a standardized platform for mechanistic and molecular analyses, including RNA isolation and gene expression (figures 4–7) [1]. Tilapia, representing an aquaculture species, was used to evaluate treatment performance under more practical conditions relevant to farming environments, where skin injuries can affect survival and productivity [43]. Using a 3 W laser-induced injury model, both species exhibited clear wound recovery. Tilapia achieved near-complete macroscopic healing within approximately 30 days (figure 2), likely owing to their high skin moisture and elevated type I collagen levels, which support scar prevention and efficient tissue repair [44]. By comparison, zebrafish required around 50 days to reach a similar level of healing (figure 3).

Despite these advantages, some limitations should be noted. Zebrafish skin lacks a fully keratinized stratum corneum and has a simpler structure than mammalian skin, which may affect barrier function and healing responses [1,45]. Additionally, the aquatic environment restricts the use of conventional topical treatments, emphasizing the need for specialized biomaterials. Overall, these models are best considered complementary, providing both mechanistic insight and practical relevance for hydrogel-based wound therapy evaluation.

### 4.2. Water immersion of the hydrogel for the wound-healing treatment

The application of the water immersion method with PVA+borax hydrogel has been highlighted to significantly enhance skin wound healing in zebrafish and tilapia (figures 2 and 3). The observed improvement may be associated with the unique properties of the hydrogel, including its ability to maintain a moist wound environment, absorb excess fluids and create a supportive microenvironment essential for cellular activities critical to wound repair. PVA+borax hydrogels possess a high fluid absorption capacity, which maintains optimal moisture levels essential for wound healing. Such a moist environment prevents scab formation, promotes effective cell migration and proliferation and accelerates tissue regeneration [46,47]. Their excellent swelling properties enable efficient absorption of wound exudates, preventing dehydration while creating ideal healing conditions [48,49]. Studies imply that hydrogels with greater swelling ratios enhance wound outcomes by regulating moisture at the injury site [50]. The physical properties of PVA+borax hydrogels, reinforced by borax cross-linking, further contribute to their effectiveness. Their self-healing capability allows structural recovery after physical stress, maintaining integrity and function throughout the healing process [51]. This property is particularly advantageous in aquatic environments, where the hydrogel must remain stable during immersion. Moreover, the hydrogel's interaction with the biological environment of zebrafish and tilapia could contribute to cellular processes essential for wound repair. By sustaining moisture and releasing bioactive molecules, it may support keratinocyte and fibroblast proliferation, key for re-epithelialization and granulation tissue formation [52,53].

### 4.3. Immune and inflammatory response profiles in treated zebrafish wounds

The qRT-PCR results at 10 dpw (figure 4) show how PVA and borax influence the immune and inflammatory environment during zebrafish skin repair. After injury, the wounded group displayed elevated expression of several immune-related genes, confirming activation of inflammatory pathways. Among these, *tnfa* was significantly increased compared with controls [54] and reduced in both PVA- and borax-treated groups, suggesting modulation of inflammatory responses during wound healing [55,56]. TNF-α is a key early inflammatory mediator involved in leukocyte recruitment and cytokine amplification during wound healing [54,57,58], so its reduction is consistent with progression towards resolution of the inflammatory response. Similarly, *arg2* was elevated in the wounded group but reduced to near-control levels in the PVA-treated group, while borax-treated fish showed intermediate expression, possibly reflecting a more progressed healing stage [59]. As ARG2 is linked to macrophage activity and arginine metabolism, these changes suggest modulation of macrophage-related responses rather than complete suppression [59–61]. Other genes showed consistent but non-significant trends. *il1b* and *cd3e* were higher in wounded fish and reduced after treatment, particularly with PVA, indicating decreased inflammatory and T cell–associated activity [57]. *cd79a* also increased after injury and tended to decrease with treatment, suggesting modulation of adaptive immune responses. By contrast, *nos2b* was elevated in treated groups, especially borax, potentially reflecting altered nitric oxide signalling involved in repair processes [62,63]. Overall, PVA and borax appear to modulate rather than suppress immune and inflammatory pathways during zebrafish wound healing.

### 4.4. Modulation of wound healing-related genes by hydrogel components in zebrafish wound healing

Following injury, the wounded group showed increased expression of *vegf*, *tgfb1* and *il10*, indicating activation of angiogenic and anti-inflammatory pathways essential for tissue repair [1,64,65]. For ECM remodelling, *mmp9* and *timp2b* were elevated in the wounded group and reduced in treated groups towards control levels; this may indicate a shift from active matrix degradation to more balanced remodelling [66]. Chemokine-related genes also showed modulation, with *ccl34b.4* upregulated after injury and reduced under treatment.

Importantly, *col4a1* and *col4a2*, which encode major components of type IV collagen in the basement membrane [67], showed significant but contrasting regulation. *col4a1* expression was significantly elevated in the borax-treated group compared with the wounded group, suggesting changes associated with basement membrane remodelling and structural support during tissue regeneration [68,69]. By contrast, *col4a2* expression was significantly reduced in both treatment groups, with a more pronounced decrease observed in the PVA-treated group. This divergence indicates that hydrogel components differentially influence collagen IV composition, potentially reflecting distinct mechanisms of basement membrane remodelling [70]. Given that type IV collagen is essential for maintaining tissue architecture and facilitating cell adhesion and migration [71,72], such selective modulation may contribute to improved wound-healing outcomes. Overall, the significant regulation of *col4a1* and *col4a2* suggests their potential involvement in hydrogel-associated wound repair. These findings suggest that PVA and borax fine-tune key components of healing rather than broadly altering transcription, supporting tissue regeneration and skin restoration.

### 4.5. PVA+borax hydrogel exhibits excellent biocompatibility suitable for skin wound-healing application

The biocompatibility of PVA+borax hydrogel for skin wound healing was evaluated using zebrafish embryos, an ideal highly sensitive model owing to their transparency and rapid development [73]. Embryos were exposed to 0.01–100 ppm hydrogel from 24 to 120 hpf. No mortality occurred in any group (supplementary material, table S6), highlighting the hydrogel's safety even at high concentrations [74,75]. This aligns with prior studies showing zebrafish assays reliably predict mammalian developmental toxicity [76,77]. Embryo viability was maintained across all concentrations, confirming the hydrogel's compatibility with biological tissues [78]. Effects of individual components—PVA, borax and their hydrogel—were also assessed (figure 8A). At 20 ppm, no structural malformations were observed, consistent with previous reports on PVA-based biomaterials [79]. All treatments caused mild hatching delays, most pronounced with borax, which also led to reduced larval growth (figure 8). This is consistent with borax-induced genotoxic stress that can interfere with chorion digestion and hatching enzyme activity [80], as reflected in smaller body size at 72–120 hpf (figure 8B) [81]. Importantly, incorporation of PVA into the hydrogel mitigated borax's adverse effects. Overall, these results demonstrate that PVA+borax hydrogel is highly biocompatible, well tolerated and safe for developing zebrafish, supporting its potential as an effective biomaterial for wound-healing applications.

### 4.6. Behavioural effects of hydrogel components on adult zebrafish

Behavioural assessments of adult zebrafish treated with PVA, borax and PVA+borax hydrogel revealed distinct effects on locomotor activity and exploratory behaviour. PVA and borax treatments may be associated with mild hypoactivity patterns, characterized by altered locomotor activity (supplementary material, figure S4A–D), consistent with stress responses in aquatic models, where exposure to aversive stimuli typically leads to reduced exploration [82,83]. By contrast, hydrogel-treated zebrafish exhibited behavioural endpoints closer to controls, indicating reduced adverse effects compared with PVA and borax groups. Additionally, borax-exposed fish showed increased alterations in meandering and fractal dimension, reflecting uncoordinated and irregular swimming patterns (supplementary material, figure S4E,G); this may raise concerns for long-term use. Conversely, zebrafish treated with PVA or hydrogel demonstrated higher movement entropy (supplementary material, figure S4H), suggesting more stable and flexible swimming behaviour and healthier neuromotor function [84,85]. Similarities in movement patterns between control and PVA+borax hydrogel groups imply minimal negative behavioural effects, supporting their use in restoring function post-injury without additional stress [86]. Overall, the behavioural alterations induced by all treatments were not markedly toxic; however, the PVA+borax hydrogel mitigated the effects observed with the individual components, bringing the endpoints closer to control levels (figure 9A,B). These results demonstrate a neutral behavioural profile, high biocompatibility and low mortality (figure 9C,D), indicating that hydrogel treatment preserves locomotor activity and exploratory behaviour. Such functional stability supports its safety for biomedical applications, particularly wound healing, where maintaining physiological and psychological function is critical [87,88].

## 5. Conclusion

This study provides a robust and reproducible methodology for evaluating wound-healing biomaterials in aquatic systems by integrating a laser-induced skin ablation model with water immersion-based treatment delivery. The precisely controlled laser injury enabled consistent full-thickness wounds in zebrafish, allowing reliable quantification of healing kinetics and direct correlation with molecular and histological outcomes, addressing key limitations in standardizing injury and treatment delivery in aquatic models.

A key novelty is the development of a simple PVA+borax hydrogel that can be administered via immersion, eliminating direct application while maintaining therapeutic efficacy. The hydrogel showed a complementary effect, with PVA and borax contributing different roles in regulating inflammation and tissue repair. qRT-PCR analysis showed lower expression of several injury-induced inflammatory and macrophage-associated markers in the treatment groups, which may suggest modulation of inflammatory responses during wound healing. In parallel, genes associated with basement membrane structure exhibited differential regulation, suggesting coordinated ECM remodelling rather than uniform deposition. Together, these observations indicate a potential transition from inflammation towards organized tissue reconstruction.

This study further employs a dual aquatic model system using zebrafish and tilapia, linking mechanistic insights with translational relevance. Zebrafish enabled detailed molecular and histological analyses, while tilapia confirmed wound-healing efficacy in a farm-relevant species. Consistent improvement in wound closure across both models highlights the scalability of the hydrogel platform. Safety evaluation confirmed high biocompatibility across developmental and adult stages, with minimal morphological, toxicological or behavioural effects. Importantly, the hydrogel reduced several adverse responses associated with borax exposure, underscoring the benefit of the combined formulation. Overall, this work provides an integrated experimental framework and an immersion-deliverable hydrogel platform for aquatic wound healing, combining methodological precision, molecular validation and cross-species applicability with promising translational potential for aquaculture and biomedical applications.

## Supplementary Material


