# Supplementary material for: Novel polyvinyl alcohol–borax hydrogel with potential to accelerate skin wound healing in aquatic models: zebrafish and tilapia
Source: R Soc Open Sci. 2026 Sep 9;13(9):252176. doi: 10.1098/rsos.252176 (PMC13561047; doi:10.1098/rsos.252176)
Supplement: Supplementary file 1 [file rsos.252176.zip › rsos.252176/rsos252176_si_001.docx]

**Figure S1. Experimental design and workflow**. **(A)** morphology assay for larvae and **(B)** adult zebrafish behavior assay.

**Figure S2. Comparative wound closure kinetics under PVA, Borax, and combined treatment in zebrafish**. Wound-closure data were analyzed using two-way ANOVA with Geisser–Greenhouse correction. Dunnett’s post hoc test was performed to compare each treatment group with the control. Data presented as mean ± SD, with sample sizes of n = 10–12. Statistical significance is defined as ns: not significant, and **P* < 0.05.

**Figure S3. Comparative wound closure kinetics under PVA, Borax, and combined treatment in tilapia.** Wound-closure data were analyzed using two-way ANOVA with Geisser–Greenhouse correction. Dunnett’s post hoc test was performed to compare each treatment group with the control. Data presented as mean ± SD, with sample sizes of n = 10–11. Statistical significance is defined as ns: not significant.

**Figure S4. Behavioral analysis of zebrafish in a 3D locomotion test across different treatment groups.** PVA-treated (green bars), Borax-treated (blue bars), Hydrogel-treated (red bars), and control (black bars) groups, with six fish per shoal. A total of 14 behavioral endpoints were measured and categorized into four groups. The Locomotor Activity Endpoints category (green section) includes **(A)** freezing time, **(B)** average speed, **(C)** rapid movement time, and **(D)** swimming time. The Movements Orientation Endpoints category (blue section) consists of **(E)** meandering and **(F)** average angular velocity. Additionally, mathematical analyses were performed for Fractal Dimension & Entropy (brown section), which includes **(G)** fractal dimension and **(H)** entropy. The Exploratory Behavior Endpoints category (purple section) includes **(I)** time in the top zone, **(J)** time in the middle zone, **(K)** time in the bottom zone, **(L)** average distance to the center of the tank, **(M)** total entries into the top zone, and **(N)** total distance traveled in the top zone. Data are presented as mean ± SEM (n = 12). Statistical analysis was performed using the Kruskal–Wallis test with Dunn's multiple comparisons test. Different letters (a, b) indicate statistically significant differences (*P* < 0.05).

**Table S1.** Application of PVA-Borax hydrogel in industrial or biomedical fields.

| **Type of PVA hydrogel** | **Application** | **Reference** |
| --- | --- | --- |
| Polyvinyl alcohol-borax (PVA-B) hydrogels | Cleaning tools of various artwork surfaces | [1] |
| Polyvinyl alcohol (PVA) – borax – polyvinylpyrrolidone (PVP) | Developed hydrogels holds great potential for application in biomedical and industrial fields | [2] |
| PVA/Borax/NaBO_3_ | Decontamination of Chemical Warfare Agents (CWA) tools | [3] |
| Polyvinyl alcohol – Borax – Agarose (PVA – B/AG) | Tools for the conservation of ancient artwork | [4] |
| PVA-Borax incorporated with curcumin | Wound dressing materials and drug delivery system | [5] |
| Polyvinyl alcohol – Borax (PVA – B) | Application requirement as strain sensor on electronic devices to future robot. | [6] |
| Polyvinyl alcohol with cellulose nanocrystal (PVA-CNC) | Soil conditioner | [7] |

**Table S2.** Summary of hydrogels’ biological effect on skin wound healing.

| **Type of PVA hydrogel** | **Model** | **Concentration** | **Biological Effects** | **Reference** |
| --- | --- | --- | --- | --- |
| Polyvinyl alcohol (PVA)/Pullulan/Poly-L/Lysine/Gelatine (P/Pu/L/G) | *In vitro* assay using cell | PVA (10% w/v), PVA (10% w/v), Poly-L-lysine (10% w/w), Gelatin (10% w/w) | Improved cell migration and proliferation in the presence of P/Pu/L/G hydrogels & Potential of P/Pu/L/G for biomedical applications such as wound dressing | [8] |
| Polyvinyl Alcohol-Iodine (PAI) Hydrogels | Cells and Mice | 8% and 3.8% | MRSA-infected open wound in Balb/c mice could be efficiently healed. | [9] |
| Polyvinyl alcohol + chitosan | Rat | 5% PVA, 0.75% chitosan and 0.25% drug | PVA-chitosan hydrogel showed faster healing of the wound made in rat dorsum | [10] |
| Polyvinyl alcohol nanofiber | Cells | 10% PVA nanofiber | PVA nanofibers for sustained antibacterial activities. They also prolonged bacterial growth inhibition. | [11] |
| Adipose-derived stem cells (ADSCs)-seeded PVA dressing (ADSCs/PVA) | Cells | 400 µL ADSCs in 1 mg of PVA | ADSCs/PVA could promote the wound healing via secreting bioactive factors from ADSCs | [12] |
| Curcumin incorporation into an oxidized cellulose nanofiber-polyvinyl alcohol hydrogel | Mice | 75.66 μg/mL and 62.33 μg/mL of curcumin.  TOCN-10PVA-Cur hydrogel released only 8.67 μg/mL in hydrogel eluted PBS | Notably, wound healing markers were expressed at the wound bed which resulted in significant wound contraction after 2 weeks of curcumin loaded hydrogel application in rat skin [excisional wound](https://www.sciencedirect.com/topics/engineering/excisional-wound) model | [5] |
| Incorporation of ZnO nanoparticles into heparinised polyvinylalcohol/chitosan hydrogels | Cells | 0, 0.5, and 1% of ZnO nanoparticle/PVA/Chitosan hydrogels | VA/CS/nZnO/Heparin hydrogel bionanocomposites can effectively protect thesurface of the wound against dehydration and exudate accumulationand while preventing bacteria growth and infections, they acceleratethe performance in wound healing process | [13] |

**Table S3.** Behavioral parameters were assessed with the Fish 3D Locomotion App (F3LA).

| **Category** | **Behavioral Parameter** | **Measurement Unit** |
| --- | --- | --- |
| **Locomotor activity** | Average speed | cm/s |
|  | Freezing movement time percentage ratio | % |
|  | Swimming movement time percentage ratio | % |
|  | Rapid movement time percentage ratio | % |
| **Movement orientation** | Average angular velocity | °/s |
|  | Meandering | °/m |
| **Exploratory behaviors** | Average distance to the center of the tank (Thigmotaxis) | cm |
|  | Total distance traveled in the top portion of the tank | m |
|  | Total entries to the top portion of the tank | Times |
|  | Time in the top portion of the tank | % |
|  | Time in the middle portion of the tank | % |
|  | Time in the bottom portion of the tank | % |
| **Other behavior endpoints** | Fractal dimension | - |
|  | Entropy | - |

**Table S4.** Time-dependent dissolution of PVA+Borax hydrogel in water. **(A)** Hydrogel mass loss over time. **(B)** Water mass changes over time.

| **Time (hr)** | (A) **Hydrogel mass (g)** | (B) **Water mass (g)** |
| --- | --- | --- |
| **0** | 20.5 | 571.1 |
| **1** | 20.3 | 567.4 |
| **2** | 19.1 | 566.2 |
| **3** | 17.7 | 566.6 |
| **4** | 15.0 | 565.3 |
| **5** | 13.3 | 565.8 |
| **6** | 11.7 | 565.1 |
| **7** | 9.1 | 564.7 |
| **8** | 6.8 | 564.3 |
| **9** | 5.1 | 563.8 |
| **10** | 3.8 | 563.6 |
| **11** | 2.4 | 563.3 |
| **12** | 0.7 | 563.0 |
| **13** | 0.0 | 562.6 |

**Table S5.** Primers and genes for qRT-PCR assay.

| **Genes** | **Primers** | **Sequences** |
| --- | --- | --- |
| TGF-b | Z TGFβ1 F | CCCAAGGAACCAGAAGTAGAAG |
|  | Z TGFβ1 R | GGATCTTCTATGGTGTGCTGAA |
| Timp2b | Z TIMP2B F | GCGTTATGTGGAGTCAGTCTT |
|  | Z TIMP2B R | CTCGATGTAGTCACACAGGTTTAT |
| MMP9 | Z MMP9 F | TTTGCCCTGATCGTGGATAC |
|  | Z MMP9 R | GGGAAACCCTCCACGTATTT |
| TNF-a | Z TNFα F | AGAAGGAGAGTTGCCTTTACCGCT |
|  | Z TNFα R | AACACCCTCCATACACCCGACTTT |
| IL-1b | Z IL-1β F | TCAAACCCCAATCCACAGAG |
|  | Z IL-1β R | TCACTTCACGCTCTTGGATG |
| CCL34A | Z CCL34A.4 F | TGCAGCTCAACCAGAAGATG |
|  | Z CCL34A.4 R | CTTTGACGCATGGAGGATTT |
| CCL34B | Z CCL34B.4 F | TCGAGTTGGAGTAACATGTG |
|  | Z CCL34B.4 R | GTACCTTTCCTTCTCTGCGTAG |
| IL-10 | Z IL-10 F | CCCTATGGATGTCACGTCATG |
|  | Z IL-10 R | CATATCCCGCTTGAGTTCCTG |
| Collagen type I | Z COL1A2 F | CTGGCATGAAGGGACACAG |
|  | Z COL1A2 R | GGGGTTCCATTTGATCCAG |
| VEGF | Z VEGF F | GGTGGCGGTCCAGAAGTT |
|  | Z VEGF R | CAGTGGAGTGTTGCGGTGA |
| b-actin | Z B ACTIN F | AATCTTGCGGTATCCACGAGACCA |
|  | Z B ACTIN R | TCTCCTTCTGCATCCTGTCAGCAA |
| Collagen type 4.1 | z col4a1 F | ATGGCTGGGCTTCACTCTGGATTG |
|  | z col4a1 R | TTCAAGACAAGAGCCAGGAGAAGCC |
| Collagen type 4.2 | z col4a2 F | GTTTTAGAGGGCCTCCTGG |
|  | z col4a2 R | CCTGGTGGACCTGAGTATCC |
| prkdc | Z prkdc F | GCCTGGCTTGTGACGTTGAC |
|  | Z prkdc R | ACTGCCACTGTGTCTTGGCT |
| cd109 | Z cd109 F | GTCTGAAGTTCTGCGCTGTG |
|  | Z cd109 R | CTCCACATTCAGCTGATACGGA |
| il2a | Z IL2A F | TGATGTAGAGGATAGTTGCATTGTTGC |
|  | Z IL2A R | GAAGTGTCCGTTGTGCTGTTCTC |
| cd3e | Z CD3e F | AAGGAAAGGTGTGTAAGGACTGCTATG |
|  | Z CD3e R | GAGCGGGAAGTGGGTTTTTG |
| cd79a | Z CD79a F | GCGAGGGTGTGAAAAACAGT |
|  | Z CD79a R | CCCTTTCTGTCTTCCTGTCCA |
| nos2b | Z NOS2b F | TGAGGCAAAATTCCACCACC |
|  | Z NOS2b R | TCTGCAACAGAGACTTTCCCA |
| arg1 | Z ARG1 F | CATCTGCCAAACAGGGCTTC |
|  | Z ARG1 R | GATCTCGTCCGCTGTTCTGC |

**Table S6.** Survival test of zebrafish larvae across a concentration range of 0.01–100 ppm PVA+Borax solution. Each experimental replicate included 12–13 larvae, and all treatments were conducted in duplicate (total n = 24–26).

|  | **Control** | | **0.01 ppm** | | **0.1 ppm** | | **1 ppm** | | **10 ppm** | | **100 ppm** | |
| --- | --- | --- | --- | --- | --- | --- | --- | --- | --- | --- | --- | --- |
| hpf | 1st rep | 2nd rep | 1st rep | 2nd rep | 1st rep | 2nd rep | 1st rep | 2nd rep | 1st rep | 2nd rep | 1st rep | 2nd rep |
| 0 | 12 | 12 | 12 | 12 | 12 | 13 | 13 | 13 | 12 | 13 | 12 | 12 |
| 24 | 12 | 12 | 12 | 12 | 12 | 13 | 13 | 13 | 12 | 13 | 12 | 12 |
| 48 | 12 | 12 | 12 | 12 | 12 | 13 | 13 | 13 | 12 | 13 | 12 | 12 |
| 72 | 12 | 12 | 12 | 12 | 12 | 13 | 13 | 13 | 12 | 13 | 12 | 12 |
| 96 | 12 | 12 | 12 | 12 | 12 | 13 | 13 | 13 | 12 | 13 | 12 | 12 |
| 120 | 12 | 12 | 12 | 12 | 12 | 13 | 13 | 13 | 12 | 13 | 12 | 12 |

**Note S1.** Interaction of PVA–borax hydrogel with FeCl₂ solution. To evaluate the interaction of the hydrogel with iron ions, the material was introduced into 500 mL of FeCl₂ solution at a final concentration of 0.315 mM. The FeCl₂ solution was prepared by dissolving 0.02 g of FeCl₂ (molecular weight: 126.75 g/mol) in 0.5 L of water, yielding a calculated concentration of 0.315 mM. Prior to hydrogel addition, an initial sample (0 h) was collected to serve as a baseline. Following hydrogel introduction, 20 mL aliquots were sequentially collected at predetermined time points of 0.5, 1, 2, 4, 6, 8, 16, 24, and 48 h. During the course of the experiment, a progressive color change of the solution was observed, transitioning from initially transparent to a light yellow hue within the first few hours, and gradually intensifying to a darker yellow/orange coloration over time (Figure S5). This change suggests interaction between the hydrogel matrix and Fe²⁺ ions in the solution. In parallel, the hydrogel gradually dissolved in the aqueous environment, with near-complete dissolution observed by 48 h. These observations indicate a time-dependent response of the hydrogel in the presence of iron ions, potentially associated with ion exchange, complex formation, or structural destabilization of the hydrogel network.

**Figure S5**. Samples were collected at the designated time points (from left to right: 0.5, 1, 2, 4, 6, 8, 16, 24, and 48 h).

**References:**

1.	Al-Emam, E.; Soenen, H.; Caen, J.; Janssens, K. Characterization of polyvinyl alcohol-borax/agarose (PVA-B/AG) double network hydrogel utilized for the cleaning of works of art. *Heritage Science* **2020**, *8*, 1-14.

2.	Huang, M.; Hou, Y.; Li, Y.; Wang, D.; Zhang, L. High performances of dual network PVA hydrogel modified by PVP using borax as the structure-forming accelerator. *Designed monomers and polymers* **2017**, *20*, 505-513.

3.	Redy Keisar, O.; Nahum, V.; Yehezkel, L.; Marcovitch, I.; Columbus, I.; Fridkin, G.; Chen, R. Active and Strippable PVA/Borax/NaBO3 Hydrogel for Effective Containment and Decontamination of Chemical Warfare Agents. *ACS omega* **2021**, *6*, 5359-5367.

4.	Al-Emam, E.; Motawea, A.G.; Janssens, K.; Caen, J. Evaluation of polyvinyl alcohol–borax/agarose (PVA–B/AG) blend hydrogels for removal of deteriorated consolidants from ancient Egyptian wall paintings. *Heritage Science* **2019**, *7*, 1-18.

5.	Shefa, A.A.; Sultana, T.; Park, M.K.; Lee, S.Y.; Gwon, J.-G.; Lee, B.-T. Curcumin incorporation into an oxidized cellulose nanofiber-polyvinyl alcohol hydrogel system promotes wound healing. *Materials & Design* **2020**, *186*, 108313.

6.	Zheng, W.; Li, Y.; Xu, L.; Huang, Y.; Jiang, Z.; Li, B. Highly stretchable, healable, sensitive double-network conductive hydrogel for wearable sensor. *Polymer* **2020**, *211*, 123095.

7.	Wang, Z.; Ding, Y.; Wang, J. Novel Polyvinyl Alcohol (PVA)/Cellulose Nanocrystal (CNC) supramolecular composite hydrogels: preparation and application as soil conditioners. *Nanomaterials* **2019**, *9*, 1397.

8.	Shitole, A.A.; Raut, P.W.; Khandwekar, A.; Sharma, N.; Baruah, M. Design and engineering of polyvinyl alcohol based biomimetic hydrogels for wound healing and repair. *Journal of Polymer Research* **2019**, *26*, 1-11.

9.	Miao, Z.; Sun, Y.; Tao, Z.; Chen, Y.; Ma, Y.; Zhu, D.; Huang, X.; Zha, Z. Thermochromic Polyvinyl Alcohol‐Iodine Hydrogels with Safe Threshold Temperature for Infectious Wound Healing. *Advanced healthcare materials* **2021**, 2100722.

10.	Sung, J.H.; Hwang, M.-R.; Kim, J.O.; Lee, J.H.; Kim, Y.I.; Kim, J.H.; Chang, S.W.; Jin, S.G.; Kim, J.A.; Lyoo, W.S. Gel characterisation and in vivo evaluation of minocycline-loaded wound dressing with enhanced wound healing using polyvinyl alcohol and chitosan. *International journal of pharmaceutics* **2010**, *392*, 232-240.

11.	Jatoi, A.W.; Ogasawara, H.; Kim, I.S.; Ni, Q.-Q. Polyvinyl alcohol nanofiber based three phase wound dressings for sustained wound healing applications. *Materials Letters* **2019**, *241*, 168-171.

12.	Gao, T.; Jiang, M.; Liu, X.; You, G.; Wang, W.; Sun, Z.; Ma, A.; Chen, J. Patterned polyvinyl alcohol hydrogel dressings with stem cells seeded for wound healing. *Polymers* **2019**, *11*, 171.

13.	Khorasani, M.T.; Joorabloo, A.; Moghaddam, A.; Shamsi, H.; MansooriMoghadam, Z. Incorporation of ZnO nanoparticles into heparinised polyvinyl alcohol/chitosan hydrogels for wound dressing application. *International journal of biological macromolecules* **2018**, *114*, 1203-1215.
